# 10-y Risks of Death and Emergency Re-admission in Adolescents Hospitalised with Violent, Drug- or Alcohol-Related, or Self-Inflicted Injury: A Population-Based Cohort Study

**DOI:** 10.1371/journal.pmed.1001931

**Published:** 2015-12-29

**Authors:** Annie Herbert, Ruth Gilbert, Arturo González-Izquierdo, Alexandra Pitman, Leah Li

**Affiliations:** 1 Population, Policy & Practice Programme, Institute of Child Health, University College London, London, United Kingdom; 2 Farr Institute of Health Informatics Research, Department of Epidemiology and Public Health, University College London, London, United Kingdom; 3 Division of Psychiatry, University College London, London, United Kingdom; Western Sydney University, AUSTRALIA

## Abstract

**Background:**

Hospitalisation for adversity-related injury (violent, drug/alcohol-related, or self-inflicted injury) has been described as a “teachable moment”, when intervention may reduce risks of further harm. Which adolescents are likely to benefit most from intervention strongly depends on their long-term risks of harm. We compared 10-y risks of mortality and re-admission after adversity-related injury with risks after accident-related injury.

**Methods and Findings:**

We analysed National Health Service admissions data for England (1 April 1997–31 March 2012) for 10–19 y olds with emergency admissions for adversity-related injury (violent, drug/alcohol-related, or self-inflicted injury; *n* = 333,009) or for accident-related injury (*n* = 649,818). We used Kaplan–Meier estimates and Cox regression to estimate and compare 10-y post-discharge risks of death and emergency re-admission. Among adolescents discharged after adversity-related injury, one in 137 girls and one in 64 boys died within 10 y, and 54.2% of girls and 40.5% of boys had an emergency re-admission, with rates being highest for 18–19 y olds. Risks of death were higher than in adolescents discharged after accident-related injury (girls: age-adjusted hazard ratio 1.61, 95% CI 1.43–1.82; boys: 2.13, 95% CI 1.98–2.29), as were risks of re-admission (girls: 1.76, 95% CI 1.74–1.79; boys: 1.41, 95% CI 1.39–1.43). Risks of death and re-admission were increased after all combinations of violent, drug/alcohol-related, and self-inflicted injury, but particularly after any drug/alcohol-related or self-inflicted injury (i.e., with/without violent injury), for which age-adjusted hazard ratios for death in boys ranged from 1.67 to 5.35, compared with 1.25 following violent injury alone (girls: 1.09 to 3.25, compared with 1.27). The main limitation of the study was under-recording of adversity-related injuries and misclassification of these cases as accident-related injuries. This misclassification would attenuate the relative risks of death and re-admission for adversity-related compared with accident-related injury.

**Conclusions:**

Adolescents discharged after an admission for violent, drug/alcohol-related, or self-inflicted injury have increased risks of subsequent harm up to a decade later. Introduction of preventive strategies for reducing subsequent harm after admission should be considered for all types of adversity-related injury, particularly for older adolescents.

## Abstract 2

In a UK cohort study, Annie Herbert and colleagues compare long-term risks between adolescents hospitalized for adversity- versus accident-related injuries.

## Editors' Summary

**Background:**

Adolescence—the period of human growth and development that occurs between the ages of 10 and 19 years—prepares the body and mind for adulthood. It is characterized by numerous, sometimes troubling, physical, mental, emotional, and social changes. Perhaps the biggest change is puberty, which usually occurs between the ages of 10 and 14 years for girls and between the ages of 12 and 16 years for boys. During puberty, several biological changes prepare the body for parenthood. For example, the breasts develop and menstrual periods begin in girls, and the testicles and penis grow in boys. Physiological growth is also rapid during puberty, and, by the end of puberty, both boys and girls are at or close to their adult height and weight. Adolescence is also the time when individuals begin to move towards social and economic independence, and when they acquire their own unique personality and opinions, the emotional skills needed to form adult relationships, and the intellectual capacity for abstract reasoning.

**Why Was This Study Done?:**

Adolescents often use alcohol and other drugs unwisely, and because adolescents do not fully understand the relationships between behavior and consequences, everyday situations sometimes escalate into violence. Moreover, some adolescents intentionally damage their body (self-harm) as a way to deal with the emotional upheaval of adolescence. Consequently, adolescents often sustain adversity-related injuries (violence-related, drug- or alcohol-related, or self-inflicted injuries). In England, for example, a third of all adolescents admitted to hospital for any injury have an adversity-related injury; the remaining injuries are mainly accident-related. Because adolescents who present with an adversity-related injury often re-present later with other adversity-related injuries, hospitalization for such injuries is a “teachable moment,” a time when relevant interventions (for example, psychosocial interventions that deal with psychological and social development) can potentially reduce the risk of further harm. But which adolescents are likely to benefit from such interventions depends on their long-term risks of harm. Here, the researchers use hospitalization data for England to compare the ten-year risks of mortality (death) and re-admission among adolescents after adversity-related injury with the risks after accident-related injury.

**What Did the Researchers Do and Find?:**

The researchers used National Health Service hospital admissions data collected between 1997 and 2012 for 10–19 year olds with emergency admissions for adversity-related or accident-related injury (333,009 and 649,818 adolescents, respectively) to estimate the ten-year risks of death and emergency re-admission among injured adolescents after discharge. Among adolescents discharged after an adversity-related injury, one in 137 girls and one in 64 boys died within ten years. Also, 54.2% of girls and 40.5% of boys had a subsequent emergency re-admission for an adversity-related injury, and emergency re-admission rates were highest in 18–19 year olds. The risks of both death and emergency re-admission were higher among adolescents discharged after an adversity-related injury than among adolescents discharged after an accident-related injury. For example, boys discharged after an adversity-related injury were about twice as likely to die within the next ten years as boys discharged after an accident-related injury. Risks of death were increased after all combinations of adversity-related injury but particularly after combinations that included drug- or alcohol-related or self-inflicted injury. Finally, the risks of emergency re-admission were highest after injuries that included self-inflicted injury in both girls and boys.

**What Do These Findings Mean?:**

These findings indicate that adolescents discharged from the hospital after an admission for violence-related, drug- or alcohol-related, or self-inflicted injuries have increased risks of subsequent harm up to a decade later. Misclassification of some adversity-related injuries as accident-related injuries may affect the accuracy of these findings. Another important limitation of this observational study is residual confounding. That is, although the researchers adjusted for known factors likely to affect the risk of re-admission or death in their analysis, the adolescents who were re-admitted or died subsequent to discharge after an adversity-related injury may have shared other unknown characteristics that were responsible for their increased risk of harm. Nevertheless, these findings identify several risk factors that clinicians and service providers can use to identify those adolescents admitted to hospital with an injury who are at high or low risk of subsequent harm. Specifically, these findings suggest that the introduction of strategies for reducing subsequent harm after discharge should be considered for all types of adversity-related injury, particularly when it occurs in older adolescents.

**Additional Information:**

This list of resources contains links that can be accessed when viewing the PDF on a device or via the online version of the article at http://dx.doi.org/10.1371/journal.pmed.1001931.

- The World Health Organization (WHO) provides brief information on adolescence (in several languages) and links to WHO documents concerned with adolescent health
- The UK Royal College of Psychiatrists provides a fact sheet on surviving adolescence for parents, teachers, and young people
- The American Academy of Pediatrics also provides information on the stages of adolescence (in English and Spanish)
- The UK not-for-profit organization Young Minds has real stories about self-harm and about other aspects of emotional well-being and mental health among young people
- MedlinePlus provides basic information and links to further resources about adolescent development, self-harm, teen violence, and injuries
- A previous open-access paper by the researchers about violence, self-harm, and drug or alcohol misuse among adolescents admitted to the hospital for injuries is available

## Introduction

Adolescents (10–19 y olds) are a vulnerable population [1]. Community surveys of adolescents in high-income countries have estimated that up to 50%–60% are exposed to violence, drug/alcohol misuse, or self-harm [2–4]. These adverse experiences are associated with underlying psychosocial difficulties [1] and tend to co-occur [5]. There is evidence that interventions that address psychosocial difficulties have the potential to improve health and social outcomes throughout the rest of the life course [1,6,7].

A presentation to hospital for injury related to violence, drug/alcohol misuse, or self-harm provides an opportunity to assess adolescents’ psychosocial needs or initiate interventions, at a time when these individuals may be willing to consider behaviour change [8–10]. Currently, national guidance in England mandates psychosocial assessment after hospital presentations for self-harm but not after presentations for violent or drug/alcohol-related injury [11]. National guidance for managing patients with violent injury does not exist, and guidelines for managing patients with drug/alcohol-related injury focus on adult patients with drug/alcohol dependence [12,13]. If adolescents presenting with these injuries are at increased risks of future harm, then psychosocial management might be appropriate.

We have previously reported the prevalence of emergency admissions for violent, drug/alcohol-related, and self-inflicted injury for adolescents in England. Approximately 4% of girls and boys have such an admission at least once between the ages of 10 and 19 y old, accounting for one-third of adolescents admitted with any injury (the majority of the remaining two-thirds are related to accidents) [5]. In England, it is estimated that among adolescents who present to hospital with self-inflicted injury, 27.3% re-present with another self-inflicted injury in the next 1–7 y [14], and at least 9.9/1,000 die in the next 1–10 y. In a recent US study of young people presenting with violent and/or drug/alcohol-related injury, 22.4%–36.7% of those surveyed within the 2 y after discharge had re-presented with a violent injury [15]. We found no published studies reporting risks of death or re-admission through any cause following all three types of adversity-related injury (violent, drug/alcohol-related, or self-inflicted injury).

We used national hospitalisation data for England to determine the cumulative risks of death and emergency re-admission in adolescents over the 10 y after discharge following an admission for violent, drug/alcohol-related, or self-inflicted injury. We determined whether risks after adversity-related injury were increased compared with after accident-related injury (our hypothesis was that they would be). We also examined whether risks of death or emergency re-admission differed by sex and age or were associated with underlying chronic conditions, ethnicity, or deprivation.

## Methods

### Study Design and Setting

We used anonymised Hospital Episode Statistics (HES) data comprising all hospital admissions to the National Health Service (NHS) in England from 1 April 1997 to 31 March 2012 [16]. We compared outcomes for adolescents admitted to the emergency department for an adversity-related injury with those admitted with an accident-related injury. HES data captured the vast majority of our population of interest, i.e., patients admitted to hospital for injury in England [17]. Therefore, we did not carry out a sample size calculation.

We identified adolescents (aged 10–19 y inclusive) who had one or more emergency (acute, unplanned) admissions for injury. We considered any multiple admissions within 1 d of each other, or relating to a hospital transfer, to be the same admission. We defined “emergency” admissions by the HES “method of admission” variable [18], and “injury” by the use of any “S” or “T” International Classification of Diseases–10th Revision (ICD-10) diagnosis code in the admission record [19]. Most adolescents (89%) had only one emergency admission for injury at age 10–19 y and between 1 April 1997 and 31 March 2012, which was defined as the index admission. For adolescents who had two or more emergency admissions for injury, we randomly selected one as the index admission. We chose to select an index admission randomly rather than using the first admission as this would better represent presentations seen in practice. Adolescents who died at the index admission, had an invalid discharge date, or were not discharged by 31 March 2012 were excluded from analyses.

We received a standard, de-identified data extract from the Health and Social Care Information Centre (HSCIC), which does not require research ethics approval or patient consent [20]. The original study design and analysis plan (at the outset of the study) and deviations from this plan are summarised in S1 Table.

### Exposure and Outcome

**Fig 1:**
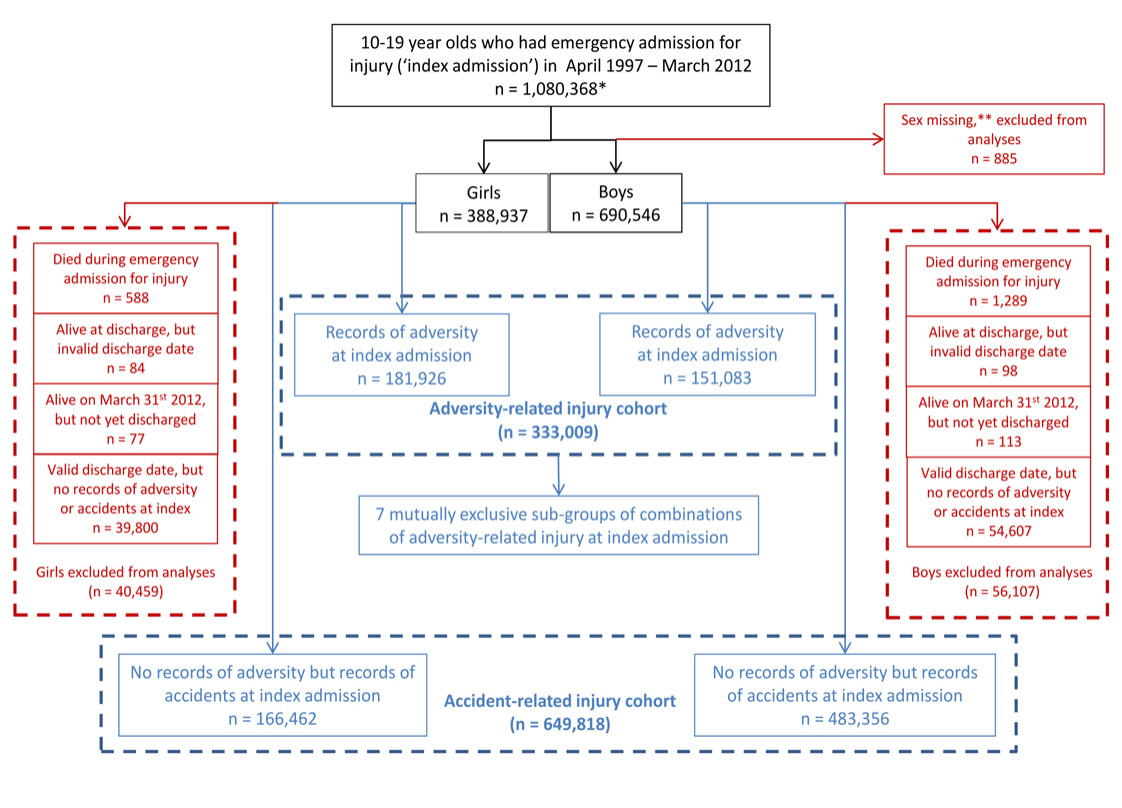
Formation of adversity-related injury and accident-related injury cohorts among 10–19 y olds. *49,784 girls and 80,205 boys had more than one emergency admission for injury between 10 and 19 y. For each of these adolescents, one emergency admission for injury was randomly selected as the index emergency admission for injury. **Not possible to impute any missing values at 0–30 y old.

We used ICD-10 codes recorded in the index admission record to define two cohorts of adolescents who were alive at discharge (Fig 1). We defined one cohort of adolescents whose index admission was for “adversity-related injury”, i.e., codes indicating any injury coupled with violence, intentional self-harm, or drug/alcohol misuse We defined drug/alcohol “misuse” as any codes indicating drug or alcohol use, since emergency admission for injury combined with drug or alcohol use indicates clinically important evidence of harm. The comparison cohort comprised adolescents whose index admission record had no codes indicating adversity-related injury but codes indicating “accident-related injury”. Codes used to define adversity and accidents are described elsewhere [5] and have been validated using hospital clinician records [21]. An admission may be categorised as being for more than one type of adversity-related injury because up to 20 ICD-10 codes can be recorded per admission. Adolescents who had an emergency admission for injury with no codes for adversity or accident were excluded from the analyses. The majority of those excluded had complex conditions or complications of surgery [5].

The outcomes were death and emergency re-admission at least 1 d after discharge from the index admission and up to 10 y later. Death was captured by linking hospital admissions to UK death registration data from the Office for National Statistics [22]. Re-admissions were captured by linking records for the same person using a HES pseudo-identifier, which was specific to our data extract. These identifiers are generated by the HSCIC using a deterministic algorithm to link all episodes of care within the English NHS using sex, date of birth, NHS number, and postcode [23]. Linkages are carried out by the HSCIC before sending out standardised data extracts.

### Confounders and Risk Factors

We included age, chronic condition status, deprivation (socioeconomic status), and ethnicity, as recorded at the index admission, as possible confounding variables, or independent risk factors, for death and re-admission, based on previous studies that have shown associations between these factors and harm in adolescents [5,24]. Other factors, such as family and neighbourhood factors, are associated with risks of future harm [25]. We did not include these confounding factors in our analyses as they are unlikely to be used by clinicians or service providers to identify groups at high risk of subsequent harm who might benefit from interventions.

We grouped adolescents into three age groups (10–14, 15–17, 18–19 y) to reflect stages of development [5]. We defined a chronic condition as any record of a chronic physical or mental condition at the index or any preceding admission based on a previously validated cluster of ICD-10 codes (excluding codes for adversity) [5]. This cluster defines a chronic condition as one requiring at least 1 y of medical treatment or follow-up [26]. We grouped the 16 ethnicity categories provided in HES into five categories (white, black, Asian, mixed, other) [27], such that groups would be large enough to stratify analyses by age and sex. Deprivation was grouped according to quintiles of Index of Multiple Deprivation (IMD) score based on residential postcode (with the least deprived areas having a score lower than the first quintile and the most deprived areas having a score higher than the fourth quintile) [18,28]. We grouped deprivation by quintile because previous research has shown differences between quintiles in the incidence of admission for violent injury [29].

We addressed data quality by replacing missing or inconsistent variables with the corresponding modal value for all admission records on the same individual. We replaced 0.8% of records with modal values for sex, 30.2% for ethnicity, and 1.8% for deprivation value. We analysed any residual missing variables for sex, ethnicity, or deprivation as “missing”. No data were missing for age or chronic condition status.

### Analyses

All analyses were carried out separately for girls and boys, given well-established differences in the frequency of adversity-related injury between the sexes [5]. We used time-to-event analysis methods (Kaplan–Meier estimates and Cox regression) to account for variation in the length of follow-up.

To determine the absolute risks of death and emergency re-admission in each cohort, we calculated Kaplan–Meier cumulative probabilities and 95% confidence intervals (CIs) for each age group from 1 d to 10 y after discharge from the index admission. We also calculated 1-, 5-, and 10-y risks of death and re-admission following any violent, any drug/alcohol-related, and any self-inflicted injury. To allow comparison of the risks of death in the adversity-related and accident-related injury cohorts with those in the general population, we derived general population estimates of risks of death in 10–19 y olds in 1997–1999 for the next 1–10 y in 1-y increments using aggregate statistics published by the Office for National Statistics (see S2 Table for details on how these numbers were derived) [30]. A comparison of risks of re-admission in our cohorts with risks for adolescents in the general population was not possible as only 12% of the general population of adolescents had an emergency admission for injury at all [5].

We tested for differences in risks of death and emergency re-admission over time between the two cohorts using Cox regression, and present hazard ratios (HRs) with 95% CIs. Models were adjusted for confounders in stages. We first estimated crude HRs between the two cohorts, and then estimated HRs adjusting for age; age and chronic condition status; and age, chronic condition status, ethnicity, and deprivation. Estimated HRs were attenuated after adjusting for age, (e.g., by 15.5% for death in girls). However, further adjustments for chronic condition status, ethnicity, and deprivation did not substantially alter age-adjusted HRs. We therefore present the main comparison of the two cohorts adjusted by age only. However, because chronic condition status and deprivation were independently associated with death and re-admission, we report the absolute 10-y risks of these outcomes after adversity-related and accident-related injury in Table A6 in S1 Text.

As different types of adversity-related injury tend to co-occur [5], we estimated age-adjusted HRs of death and emergency re-admission for seven mutually exclusive combinations of violent, drug/alcohol-related, and self-inflicted injury (all versus accident-related injury). To determine whether there were additional risks of multiple emergency re-admissions for adolescents with adversity-related injury, we also estimated age-adjusted HRs of a second, third, fourth, and fifth emergency re-admission (less than 5% of adolescents had more than five re-admissions).

We tested the goodness of fit of the Cox regression models by plotting the Nelson–Aalen estimate of the cumulative hazard function against Cox–Snell residuals [31]. Analyses were conducted in Stata/SE 12 (StataCorp).

## Results

### Study Population

Of the 1,080,368 adolescents who had an emergency admission for injury, nearly one-third (*n* = 333,009) formed the adversity-related injury cohort, and 60% (*n* = 649,818) formed the accident-related injury cohort (Fig 1). The remaining 9% were excluded (0.2% who died at the index admission, 0.04% who either had an invalid discharge date or were not discharged by 31 March 2012, and 8.7% who were admitted with other causes of injury).

There were similar numbers of girls and boys in the adversity-related injury cohort, but boys outnumbered girls by 2:1 in the accident-related injury cohort (girls: 166,462, boys: 483,356). Compared with the accident-related injury cohort, adolescents in the adversity-related injury cohort were on average older at their index admission, more likely to have a chronic condition, and more likely to be from the most deprived areas according to IMD score (Table 1). The most common chronic conditions were chronic respiratory disorders (e.g., asthma), affecting 39.8% to 55.4% of the girls and boys with either adversity- or accident-related injury who also had a chronic condition (Table A1 in S1 Text). Mental health or behavioural disorders (that were not already in the definition for “adversity”) affected 33.0% to 33.5% of the girls and boys with an adversity-related injury and a chronic condition, but only 9.0% to 12.3% of the girls and boys with an accident-related injury and a chronic condition.

**Table 1:** Characteristics at discharge from index emergency admission for injury.

Characteristic	Girls		Boys	
	Adversity-Related Injury	Accident-Related Injury	Adversity-Related Injury	Accident-Related Injury
**All**	181,926 (100%)	166,462 (100%)	151,083 (100%)	483,356 (100%)
**Age**				
10–14 y old	47,926 (26.3%)	103,215 (62.0%)	24,301 (16.1%)	259,862 (53.8%)
15–17 y old	84,605 (46.5%)	36,624 (22.0%)	57,706 (38.2%)	137,044 (28.4%)
18–19 y old	49,395 (27.2%)	26,623 (16.0%)	69,076 (45.7%)	86,450 (17.9%)
**History of a chronic condition**	27,922 (15.3%)	18,934 (11.4%)	21,161 (14.0%)	49,436 (10.3%)
**Ethnicity** *				
White	144,522 (79.4%)	129,248 (77.6%)	109,307 (72.3%)	352,614 (73.0%)
Black	4,284 (2.4%)	3,320 (2.0%)	4,486 (3.0%)	9,917 (2.1%)
Asian	6,432 (3.5%)	4,066 (2.4%)	4,563 (3.0%)	13,633 (2.8%)
Mixed	2,448 (1.3%)	1,470 (0.9%)	1,540 (1.0%)	4,171 (0.9%)
Other	3,309 (1.8%)	2,541 (1.5%)	3,000 (2.0%)	7,491 (1.5%)
Missing	20,931 (11.5%)	25,817 (15.5%)	28,187 (18.7%)	95,530 (19.8%)
**Deprivation based on IMD score** *				
Least deprived	22,309 (12.3%)	29,002 (17.4%)	16,991 (11.2%)	85,304 (17.6%)
Second least deprived	24,941 (13.7%)	29,872 (17.9%)	19,474 (12.9%)	85,052 (17.6%)
Middle	30,698 (16.9%)	30,472 (18.3%)	24,450 (16.2%)	87,512 (18.1%)
Second most deprived	40,721 (22.4%)	32,670 (19.6%)	33,461 (22.1%)	95,821 (19.8%)
Most deprived	61,161 (33.6%)	41,923 (25.2%)	53,437 (35.4%)	122,749 (25.4%)
Missing	2,096 (1.2%)	2,523 (1.5%)	3,270 (2.2%)	6,918 (1.4%)
**Type of adversity-related injury**				
Any violent	13,262 (7.3%)		70,594 (46.7%)	
Any drug/alcohol-related	163,888 (90.1%)		85,421 (56.5%)	
Any self-inflicted	131,739 (72.4%)		44,621 (29.5%)	
**Emergency admissions prior to index (at 10–19 y old)**				
Adversity-related injury	18,311 (10.1%)	1,566 (0.9%)	8,121 (5.4%)	3,262 (0.7%)
Accident-related injury (no adversity)	5,438 (3.0%)	6,264 (3.8%)	10,328 (6.8%)	36,320 (7.5%)

Data are given as *n* (percent).

*Missing values were replaced with the modal value of admission records for that individual at 10–19 y old. If the value was still missing, it was replaced with the modal value of records for that individual at 0–30 y old.

In the adversity-related injury cohort, girls were admitted predominantly for drug/alcohol-related (90.1%) or self-inflicted (72.4%) injury, while boys were most often admitted for violent (46.7%) or drug/alcohol-related (56.5%) injury. The distribution of ethnicity did not differ substantially between the two cohorts. The median follow-up time from the index admission ranged from 6.8 to 7.7 y in both cohorts (Table A2 in S1 Text).

### Risk of Death

There were 4,782 deaths within 10 y of discharge (2,415 after adversity-related injury; 2,367 after accident-related injury) (Table A2 in S1 Text). There were twice as many deaths during the 10 y after discharge as during hospitalisation for the index admission: 71.8% of all deaths between the index admission date and 10 y later occurred after discharge from the index admission. The average time to death post-discharge in the two cohorts ranged from 3.1 y for boys admitted with accident-related injury to 4.1 y for girls admitted with accident-related injury.

**Fig 2:**
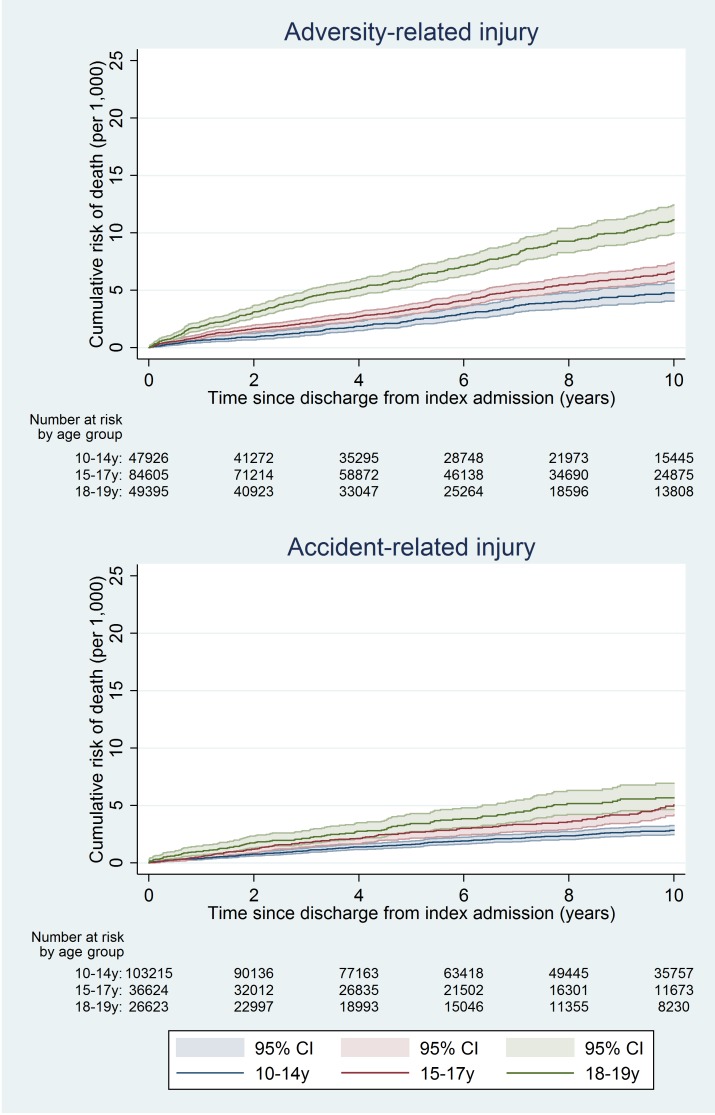
Cumulative risk of death in girls, by age group.

**Fig 3:**
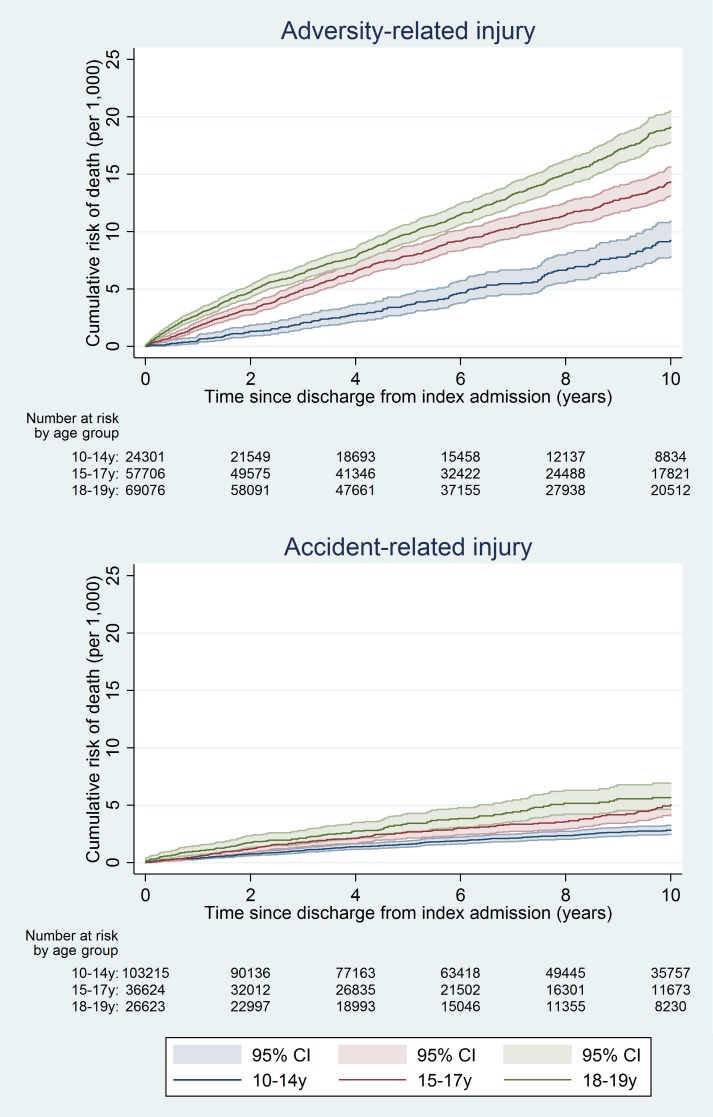
Cumulative risk of death in boys, by age group.

**Fig 4:**
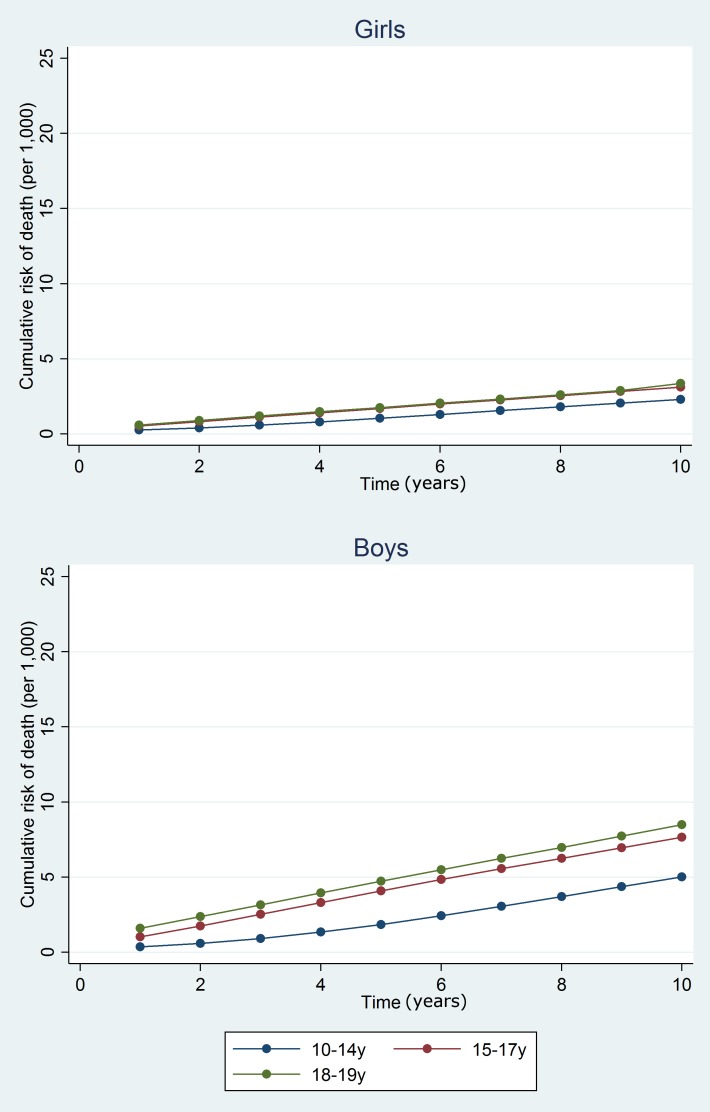
Estimated cumulative risk of death for girls and boys in the general population, by age group.

At 10 y, the cumulative risk of death after hospital discharge in the adversity-related injury cohort was 7.3/1,000 for girls (equivalent to one in 137; 95% CI, one in 147 to one in 128) and 15.6/1,000 for boys (one in 64; 95% CI, one in 68 to one in 61), compared with 3.7/1,000 girls (one in 270; 95% CI, one in 294 to one in 244) and 6.0/1,000 boys (one in 167; 95% CI, one in 175 to one in 159) in the accident-related injury cohort (Table A3 in S1 Text). Risks of death after discharge were higher following adversity-related injury than accident-related injury at all time points (Figs 2 and 3), and risks after either adversity-related or accident-related injury were higher than in the general population (Fig 4). Among girls and boys, the age group with the highest risk of death was 18–19 y olds (Figs 2 and 3). For girls in this age group, one in 90 died by 10 y after discharge from an admission for an adversity-related injury compared with one in 175 after an accident-related injury (Table A3 in S1 Text). The corresponding figures for boys in this age group were one in 52 and one in 115.

**Fig 5:**
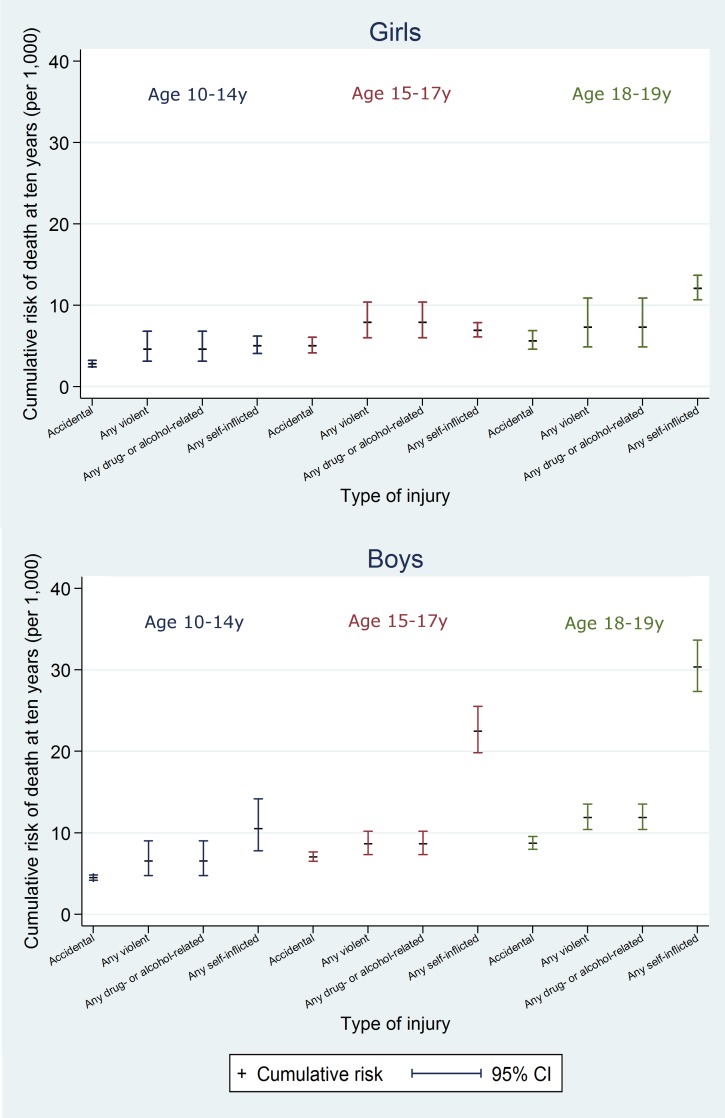
10-y risk of death by type of injury.

On average over the 10 y after discharge, risks of death in the adversity-related injury cohort compared with the accident-related injury cohort were 61% (95% CI 43%–82%) higher in girls and 113% (95% CI 98%–129%) higher in boys, after adjusting for age (95% CIs greater than unity; Table 2). Risks of death were increased in girls and boys after all combinations of violent, drug/alcohol-related, and self-inflicted injury, compared with accident-related injury (age-adjusted HRs 1.09 to 5.35; Figs 5 and S1). These risks were highest after combinations of adversity-related injury that included drug/alcohol-related injury (age-adjusted HRs: 1.61 to 5.35), though not statistically significantly for girls admitted for all three types of adversity-related injury (age-adjusted HR 2.43, 95% CI 0.91–6.51). Results of models adjusted for clinically relevant variables selected a priori are presented in Table A4 in S1 Text.

**Table 2:** Relative risks of death and emergency re-admission within 10 y of index admission.

Sex and Variable at Index	HR* (95% CI)	
	Death	Emergency Re-admission
**Girls**		
Adversity-related (versus accident-related) injury	1.61 (1.43–1.82)	1.76 (1.74–1.79)
Age group 15–17 y (versus 10–14 y)	1.46 (1.27–1.69)	1.22 (1.20–1.23)
Age group 18–19 y (versus 10–14 y)	2.32 (2.01–2.68)	1.29 (1.27–1.31)
**Boys**		
Adversity-related (versus accident-related) injury	2.13 (1.98–2.29)	1.41 (1.39–1.43)
Age group 15–17 y (versus 10–14 y)	1.68 (1.54–1.84)	1.14 (1.13–1.15)
Age group 18–19 y (versus 10–14 y)	2.16 (1.98–2.37)	1.26 (1.24–1.27)

*HRs estimated from Cox regression models, where independent variables (adversity-related/accident-related injury and age group) were entered simultaneously.

### Risk of Emergency Re-admission

There were 621,050 emergency re-admissions in both cohorts in total (Table A2 in S1 Text). On average, adolescent girls and boys in the adversity-related injury cohort had their first emergency re-admission 586 and 750 d, respectively, after discharge from the index admission, 6 and 12 mo sooner than for the accident-related injury cohort.

**Fig 6:**
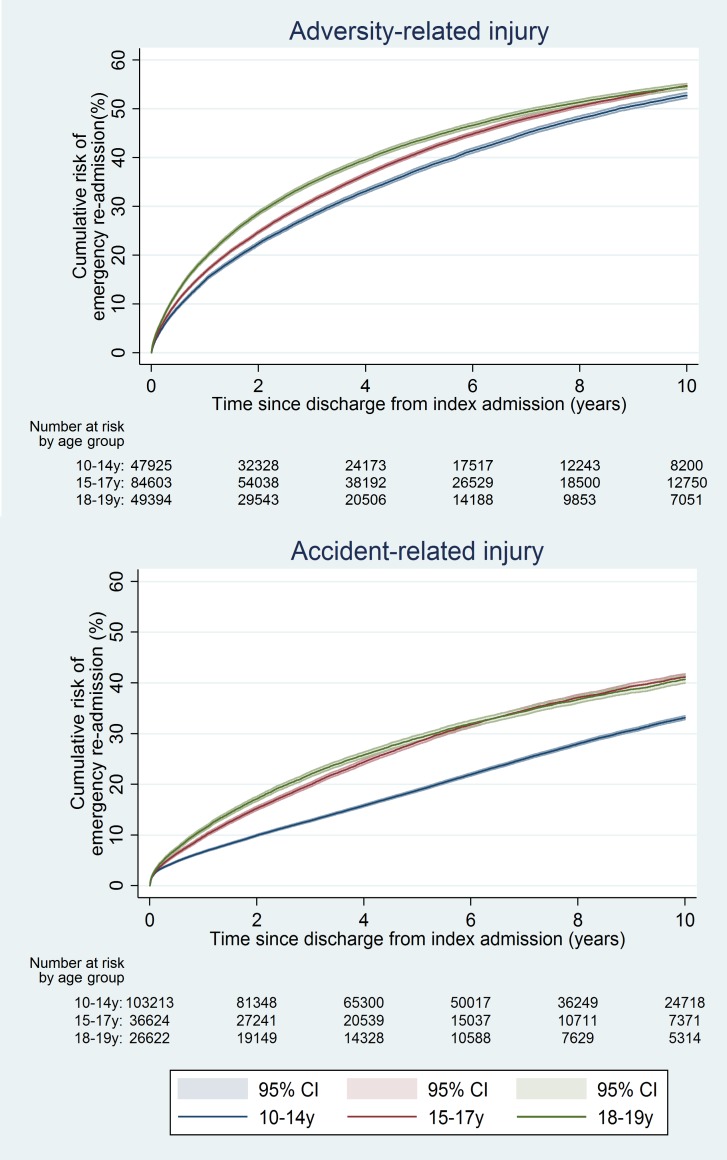
Cumulative risk of emergency re-admission in girls, by age group.

**Fig 7:**
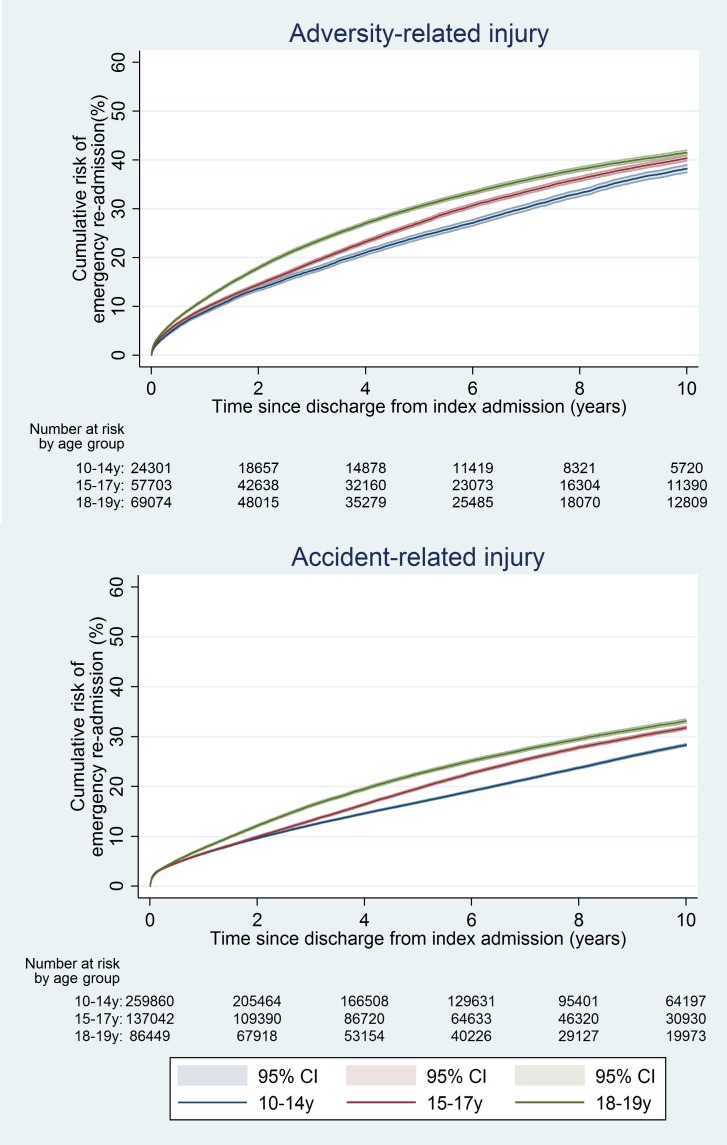
Cumulative risk of emergency re-admission in boys, by age group.

**Fig 8:**
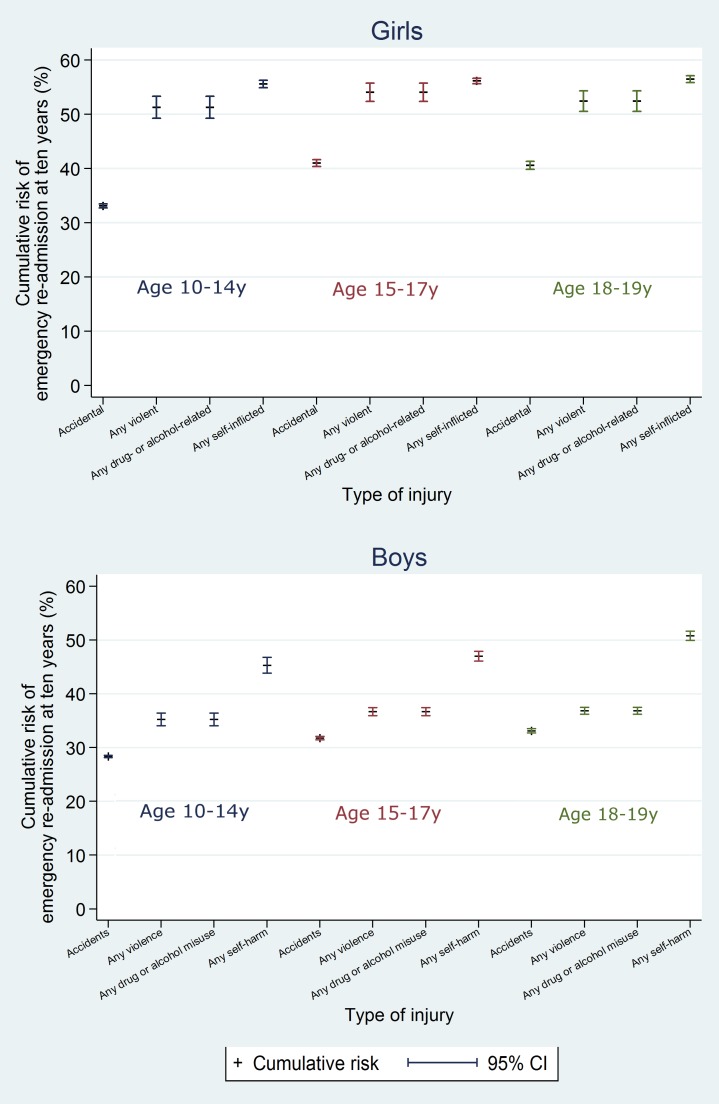
10-y risk of emergency re-admission by type of injury.

The 10-y risk of emergency re-admission was 54.2% for girls (95% CI 53.9%–54.5%) and 40.5% for boys (95% CI 40.2%–40.9%) (Table A5 in S1 Text). The cumulative risk of emergency re-admission was higher in the adversity-related injury cohort than in the accident-related injury cohort at all time points for all age–sex groups (Figs 6 and 7). In contrast to the patterns observed for cumulative risk of death, girls had a higher risk of emergency re-admission than boys for all age groups in both cohorts at all time points. The 10-y risks of emergency re-admission were higher after all types of adversity-related injury than risks in the accident-related injury cohort, for both sexes; in boys, risks were highest after self-inflicted injury (Fig 8).

By 10 y after discharge, the risk of emergency re-admission was 76% higher for girls and 41% higher for boys in the adversity-related injury cohort than for those in the accident-related injury cohort (95% CIs for age-adjusted HRs greater than unity; Table 2). Risks of emergency re-admission were highest after an injury that included self-inflicted injury in both girls and boys (age-adjusted HRs 1.29 to 3.10; S1 Fig). Results of models adjusted for clinically relevant variables selected a priori are presented in Table A4 in S1 Text.

### Risk of Multiple Emergency Re-admissions

Compared with the accident-related injury cohort, adolescents in the adversity-related injury cohort were more likely to have multiple emergency re-admissions after discharge (girls: 23.2% versus 11.64%, boys: 13.4% versus 7·0%; Table A2 in S1 Text) and were more likely to have a higher number of re-admissions (Table A6 in S1 Text). This was the case for all combinations of adversity-related injury, after adjustment for age, in both girls and boys.

### Independent Risk Factors

Relative risks of death and emergency re-admission for adversity-related injury (versus accident-related injury, estimated from Cox regression models), adjusted for age, chronic condition status, ethnicity, and deprivation are presented in Table A4 in S1 Text. In both the adversity-related and accident-related injury cohorts, presence of a chronic condition increased the risks of death and emergency admission by 2- to 5-fold in girls and boys, and girls and boys living in the most deprived areas based on IMD score had the highest risks of death and emergency re-admissions. This association persisted across all deprivation levels for deaths in boys and for emergency admissions in girls and boys (Table A7 in S1 Text).

We found no association between ethnicity and long-term risks of death or emergency re-admission after adjusting for deprivation and underlying chronic condition (Table A4 in S1 Text). Missing information on ethnicity or deprivation was associated with reduced risks of death and emergency re-admissions in girls and boys, possibly because of a failure to link recurrent admissions for individuals with incomplete data.

## Discussion

We report cumulative risks of death and emergency re-admission in adolescents in the 10 y after discharge from an admission for adversity-related injury versus accident-related injury. We found increased long-term risks of death and re-admission among adolescents discharged after hospitalisation for violent, drug/alcohol-related, or self-inflicted injury, compared with those discharged after accident-related injury. Risks of harm after both adversity-related and accident-related injury were higher than for adolescents in the general population. In the 10 y after discharge following an adversity-related injury, one in 137 girls and one in 64 boys died across all adolescent age groups. These risks for 18–19 y olds were one in 52 and one in 90. However, risks of death and re-admission were highest after any drug/alcohol-related injury or self-inflicted injury for both sexes. Having a chronic condition (typically respiratory or mental health/behavioural disorders for adolescents in this study) and living in the most deprived areas (based on residential postcode) increased the risks of death and re-admission.

### Strengths and Limitations

One strength of our study is the use of administrative data to study the entire population of adolescents hospitalised with injury within the English publicly provided NHS. Second, linkage of all hospital admissions and death records made it possible to study outcomes 10 y after discharge from the index admission. Third, this is the first study to our knowledge that has quantified risks of harm in adolescents after all three of violent, drug/alcohol-related, and self-inflicted injury within the same cohort. Long-term follow-up has previously been reported only after self-inflicted injury [14].

One limitation of our study is potential misclassification of violent, drug/alcohol-related, or self-inflicted injury as accident-related injury [32,33], which would likely lead to underestimation of the increased risk associated with adversity-related injury. For example, a study in one US hospital site showed that for up to 25% of cases of violent injury in children where violence was recorded in the medical notes, violence was not coded in the electronic discharge records [34]. Misclassification as accident-related injury may be more likely in boys, for whom behaviours such as violence or misuse of alcohol may be normalised and less likely to be noted in hospital records than for girls [35].

Another limitation is that linkage error may lead to a failure to capture death or re-admission and is more common in certain ethnic minorities. For example, in a study where the HES linkage algorithm was applied in another routine paediatric dataset, children and adolescents with black or “other” (i.e., not white, black, Asian, or mixed) ethnicity were approximately 2.4 to 4.1 times more likely to have a false or missed match than their white counterparts [36]. Failure to link events would favour underestimation of the risks of death and re-admission for these groups.

Finally, HES data do not include information on interventions received by the adolescents during or after admissions, such as referral to mental health services. Effective interventions could have confounded the association between adversity-related injury and later harm. This confounding could have caused underestimation of the association between adversity-related injury and later harm if adolescents in the adversity group were more likely to receive effective interventions than those in the accident-related injury group. In practice, however, psychosocial assessments are routinely recommended only for adolescents with self-inflicted injury [11], and it is estimated that only 60% actually receive such an assessment [37].

### Generalisability of the Study and Comparison with Other Findings

Our estimates of the relative increase in risks of death and emergency re-admission are likely to be generalisable to other hospitalised adolescent populations within the UK. However, generalisability to other countries and healthcare systems depends on the similarities of their populations of adolescents admitted with adversity-related injury to that of England. Generalisability also depends on the availability and intensity of psychosocial support for vulnerable adolescents after hospital discharge. Patterns of self-harm and drug/alcohol use during adolescence in other parts of Europe [4,38], but not in the US [39,40], have been shown to be similar to those in England.

Mortality rates in this study were either similar to or slightly higher than those reported for 14–24 y olds presenting with violent injury to the emergency department in the US [15]. The risk of violent death 2 y after a violent or drug/alcohol-related injury presentation (including non-admission) was 0.8/1,000. This figure is consistent with the risk of all causes of death 1 y after violent injury in our study (girls: 0.5/1,000; boys: 1.2/1,000). A prospective cohort study in England reported a mortality rate of 10/1,000 in 10–18 y olds followed for a median time period of 6 y after presentation to hospital for self-harm [14]. Our risks of mortality at 5 y after self-inflicted injury were similar (girls: 4.0/1,000; boys: 12.7/1,000).

### Implications for Policy, Practice, and Research

Adolescents discharged from hospital after an adversity-related injury have substantially increased risks of death and emergency re-admission during the next 10 y compared with adolescents discharged after an accident-related injury; 10 y after discharge from hospital in England for an adversity-related injury, one in 52 boys and one in 90 girls aged 18–19 y will have died. These risks may be underestimated because of under-recording of adversity in hospital discharge records.

The risk of future harm was increased after all types of adversity-related injury. These findings justify extending national policy for psychosocial assessment after self-inflicted injury to all types of adversity-related injury. Consideration of psychological and social circumstances is good clinical practice, particularly for vulnerable adolescents. However, extending mandated psychosocial assessment from self-inflicted injury to all three types of adversity-related injury may have implications for services, as injured young people aged 16 y or older are often managed on adult surgical wards in the NHS, where expertise in psychosocial assessment and support for young people may be limited. Those who are male, older, have an underlying chronic condition, or are from deprived areas, and those exposed to multiple types adversity (e.g., drug/alcohol misuse and self-harm) have the highest risks of future harm (Table A7 in S1 Text; S1 Fig). Whether interventions should be targeted at these groups requires evidence of the effectiveness, feasibility, and cost-effectiveness of interventions.

There is a lack of evidence regarding effective interventions to reduce the risk of future harm in adolescents exposed to adversity-related injury. Our confirmation that there are increased long-term risks for these adolescents highlights the need to develop and evaluate interventions. Although some interventions in the UK for self-inflicted injury have shown positive effects, these were in samples too small to provide conclusive results [3]. Brief psychosocial interventions to address violent behaviour or alcohol misuse [41–47] have shown promise for improving outcomes and for cost-effectiveness [41,48,49], but follow-up was limited to 18 mo. In addition, these interventions have been evaluated predominantly in the US, where there are cultural differences in violence and drinking behaviours, healthcare systems, and social welfare support.

## Supporting Information

S1 STROBE checklist Items that should be included in reports of observational studies. (DOC)

S1 Fig Relative risks of death and emergency re-admission within 10 y of index admission, by combinations of types of adversity-related injury. (TIF)

S1 Table Original study’s design and analysis plan and deviations from this plan for final study report. (DOCX)

S2 Table Derivation of 1- to 10-y risks of death in the general adolescent population. (DOC)

S1 Text Additional tables. (DOC)

## Abbreviations

CI: confidence interval
HES: Hospital Episode Statistics
HR: hazard ratio
HSCIC: Health and Social Care Information Centre
ICD-10: International Classification of Diseases–10th Revision
IMD: Index of Multiple Deprivation
NHS: National Health Service
