# Supplementary material for: 10-y Risks of Death and Emergency Re-admission in Adolescents Hospitalised with Violent, Drug- or Alcohol-Related, or Self-Inflicted Injury: A Population-Based Cohort Study
Source: PLoS Med. 2015 Dec 29;12(12):e1001931. doi: 10.1371/journal.pmed.1001931 (PMC4699823; doi:10.1371/journal.pmed.1001931)
Supplement: S1 STROBE checklist — (DOC) [file pmed.1001931.s001.doc]

**S1 STROBE Checklist. Items that should be included in reports of observational studies***

|  | Item No | Recommendation |  | |
| --- | --- | --- | --- | --- |
| **Title and abstract** | 1 | **(*a*) Indicate the study’s design with a commonly used term in the title or the abstract**  The title contains the phrase *“a Population-based Cohort Study”*. |  | |
|  |  | **(*b*) Provide in the abstract an informative and balanced summary of what was done and what was found**  The abstract explains the dataset used, the cohort selected, how outcomes of re-admission and death were captured, and what the ten-year risks after adversity-related and accident-related injury were, as well as the sub-groups of adolescents for which these risks were highest. |  | |
| Introduction | | | |  |
| Background/rationale | 2 | **Explain the scientific background and rationale for the investigation being reported**  Lines 52-67 provide background information. Lines 68-80 explain the rationale for this study. |  | |
| Objectives | 3 | **State specific objectives, including any pre-specified hypotheses**  Our objectives, and hypothesis that risks would be increased after adversity-related injury, are provided in lines 81-87. |  | |
| Methods | | | |  |
| Study design | 4 | **Present key elements of study design early in the paper**  The study design is described at the beginning of the Methods section in lines 91-94. |  | |
| Setting | 5 | **Describe the setting, locations, and relevant dates, including periods of recruitment, exposure, follow-up, and data collection**  The setting, location and relevant dates are described in lines 91-94. Exposures and outcomes are described in lines 116-125 and 138-139, respectively. Lines 111-113 state how the data extract was received the data extract. |  | |
| Participants | 6 | **(*a*) *Cohort study*—Give the eligibility criteria, and the sources and methods of selection of participants. Describe methods of follow-up**  Lines 98-99 state methods of selection/inclusion criteria. Lines 109-110 and 127-128 state exclusion criteria. Lines 139-142 describe how participants were followed up in Hospital Episode Statistics (HES) and Office for National Statistics Mortality (ONS) data. |  | |
|  |  | **(*b*) *Cohort study*—For matched studies, give matching criteria and number of exposed and unexposed.**  Not applicable. Adolescents in the adversity-related and accident-related injury cohorts were not matched. |  | |
| Variables | 7 | **Clearly define all outcomes, exposures, predictors, potential confounders, and effect modifiers. Give diagnostic criteria, if applicable**  Outcomes are clearly defined in lines 138-139. Exposures and outcomes are described in lines 116-125 and 138-139, respectively. Potential confounders are defined in lines 148-155. We did not analyse any factors in this study that we considered to be predictors or effect modifiers. |  | |
| Data sources/ measurement | 8* | **For each variable of interest, give sources of data and details of methods of assessment (measurement). Describe comparability of assessment methods if there is more than one group**  Each variable was recorded in HES or ONS mortality data. We made it clear that this was the case in lines 116-117, lines 139-142, and lines 148-149. There were two cohorts that were compared: it is clear in the manuscript (lines 116-117, as cited above) that each of these cohorts were defined using ICD-10 codes in HES. The fact that clusters of ICD-10 codes for adversity-related injury might be only moderately sensitive is discussed in lines 343-350. |  | |
| Bias | 9 | **Describe any efforts to address potential sources of bias.**  Lines 176-178: *“We used time-to-event analysis methods (Kaplan-Meier [K-M] estimates and Cox regression), to account for variation in the length of follow-up.”* |  | |
| Study size | 10 | **Explain how the study size was arrived at.**  Lines 84-97: *“HES data captured the vast majority of our population of interest. That is, patients admitted to hospital for injury in England. Therefore, we did not carry out any formal sample size calculation.”* |  | |
| Quantitative variables | 11 | **Explain how quantitative variables were handled in the analyses. If applicable, describe which groupings were chosen and why.**  All variables in this study were quantitative (exposures, outcomes and confounding variables that we adjusted for). We describe how we produced Kaplan Meier estimates and curves and Cox models in lines 179-212. We describe how and why age, ethicnity and deprivation were grouped in lines 156-168. |  | |
| Statistical methods | 12 | **(*a*) Describe all statistical methods, including those used to control for confounding**  All statistical methods used are described in lines 175-213. |  | |
|  |  | **(*b*) Describe any methods used to examine subgroups and interactions**  Lines 175-176: *“All analyses were carried out separately for girls and boys, given well-established differences in the frequency of adversity-related injury between sexes.”* All other subgroup analyses are described in lines 200-203. We did not examine interactions. |  | |
|  |  | **(*c*) Explain how missing data were addressed.**  We describe how missing values for confounding variables were handled in lines 169-173. We address censored outcomes in lines 176-178. |  | |
|  |  | **(*d*) *Cohort study*—If applicable, explain how loss to follow-up was addressed.**  Lines 176-178: *“We used time-to-event analysis methods (Kaplan-Meier [K-M] estimates and Cox regression), to account for variation in the length of follow-up.”* |  | |
|  |  | **(*e*) Describe any sensitivity analyses.**  We did not carry out any sensitivity analyses. |  | |
| Results | | | |  |
| Participants | 13** | **(a) Report numbers of individuals at each stage of study—eg numbers potentially eligible, examined for eligibility, confirmed eligible, included in the study, completing follow-up, and analysed**  Fig 1 is a flowchart of these numbers. The numbers of individuals still in the study at two-year time-points are provided underneath the curves in Fig 2 and in Table A3 in S1 Text. | | |
|  |  | **(b) Give reasons for non-participation at each stage.**  These have been provided in Fig 1. | | |
|  |  | **(c) Consider use of a flow diagram.**  We provide a flow diagram (Fig 1). | | |
| Descriptive data | 14** | **(a) Give characteristics of study participants (eg demographic, clinical, social) and information on exposures and potential confounders**  Summary statistics on these characteristics are provided in Table 1. | | |
|  |  | **(b) Indicate number of participants with missing data for each variable of interest**  These are provided in Table 1. | | |
|  |  | **(c) Cohort study—Summarise follow-up time (eg, average and total amount).**  Summary statistics on follow-up time are presented in Table A2 in S1 Text. The numbers of individuals still in the study at two-year time-points are provided underneath the curves in Fig 2 and in Table A3 in S1 Text. | | |
| Outcome data | 15** | ***Cohort study*—Report numbers of outcome events or summary measures over time.**  Numbers of death and re-admissions are presented in Table A2 in S1 Text. The numbers of individuals still in the study at two-year time-points are provided underneath the curves in Fig 2 and in Table A3 in S1 Text. | | |
| Main results | 16 | **(*a*) Give unadjusted estimates and, if applicable, confounder-adjusted estimates and their precision (eg, 95% confidence interval). Make clear which confounders were adjusted for and why they were included.**  Unadjusted estimates are provided in Table 2. Confounder-adjusted estimates are provided in Table A4 in S1 Text (clearly labelled by which adjustments were made). The reasons for adjustment are provided as lines 148-155 and 198-200. | | |
|  |  | **(*b*) Report category boundaries when continuous variables were categorized**  Age and deprivation scores were categorised. The category boundaries are stated in all tables that they appear (Tables 1 and 2, Figs 2 and 3, Tables A3-A5 and A7 in S1 Text). | | |
|  |  | **(*c*) If relevant, consider translating estimates of relative risk into absolute risk for a meaningful time period.**  Absolute risks are provided in Figs 2 and 3, Tables A3, A5 and A7 in S1 Text. | | |
| Other analyses | 17 | **Report other analyses done—eg analyses of subgroups and interactions, and sensitivity analyses**  The results of sex and age sub-groups are reported throughout the Results section. Absolute risks by chronic condition status and deprivation are reported in Table A7 in S1 Text. | | |
| Discussion | | | |  |
| Key results | 18 | **Summarise key results with reference to study objectives**  Key results, parallel to the three aims stated at the end of the Introduction section are described in lines 328-340. | | |
| Limitations | 19 | **Discuss limitations of the study, taking into account sources of potential bias or imprecision. Discuss both direction and magnitude of any potential bias.**  Limitations are discussed in lines 350-373. The directions and magnitudes of potential bias are discussed in lines 351-357, 359-364, and 368-371. | | |
| Interpretation | 20 | **Give a cautious overall interpretation of results considering objectives, limitations, multiplicity of analyses, results from similar studies, and other relevant evidence**  An overall interpretation is provided in this way in lines 393-398. | | |
| Generalisability | 21 | **Discuss the generalisability (external validity) of the study results**  Generalisability of results to the rest of the UK, other parts of Europe, and the US is discussed in lines 375-382. | | |
| Other information | | | |  |
| Funding | 22 | **Give the source of funding and the role of the funders for the present study and, if applicable, for the original study on which the present article is based**  A funding statement is provided in lines 587-592. | | |

*Line numbers refer to those in the final manuscript (without tracked changes).

**Give information separately for cases and controls in case-control studies and, if applicable, for exposed and unexposed groups in cohort and cross-sectional studies.

**Note:** An Explanation and Elaboration article discusses each checklist item and gives methodological background and published examples of transparent reporting. The STROBE checklist is best used in conjunction with this article (freely available on the Web sites of PLoS Medicine at http://www.plosmedicine.org/, Annals of Internal Medicine at http://www.annals.org/, and Epidemiology at http://www.epidem.com/). Information on the STROBE Initiative is available at www.strobe-statement.org.
