# Supplementary figures and images for: 10-y Risks of Death and Emergency Re-admission in Adolescents Hospitalised with Violent, Drug- or Alcohol-Related, or Self-Inflicted Injury: A Population-Based Cohort Study
Source: PLoS Med. 2015 Dec 29;12(12):e1001931. doi: 10.1371/journal.pmed.1001931 (PMC4699823; doi:10.1371/journal.pmed.1001931)

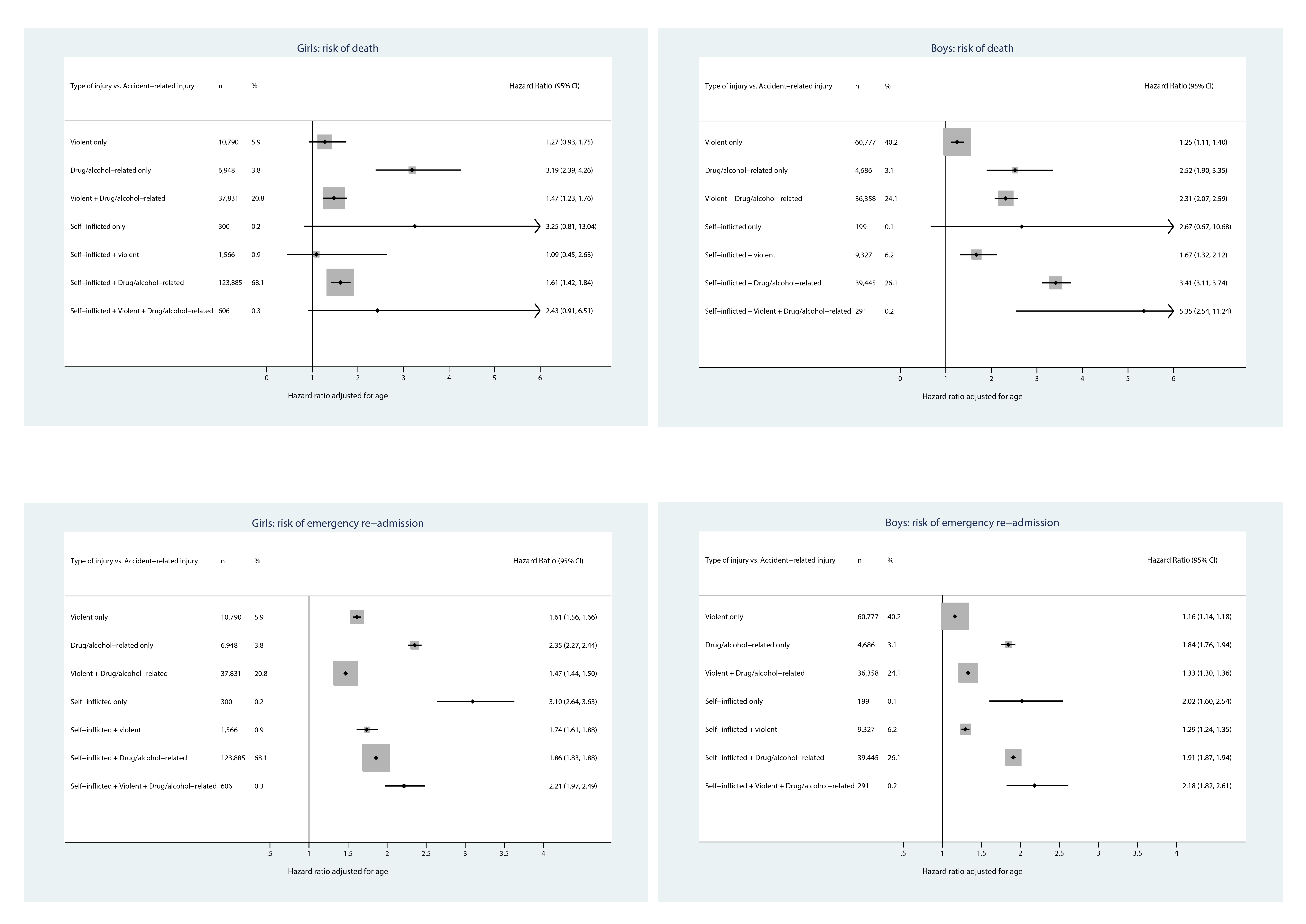

Supplement: S1 Fig — (TIF) [file pmed.1001931.s002.tif]
