# Supplementary material for: 10-y Risks of Death and Emergency Re-admission in Adolescents Hospitalised with Violent, Drug- or Alcohol-Related, or Self-Inflicted Injury: A Population-Based Cohort Study
Source: PLoS Med. 2015 Dec 29;12(12):e1001931. doi: 10.1371/journal.pmed.1001931 (PMC4699823; doi:10.1371/journal.pmed.1001931)
Supplement: S1 Table — (DOCX) [file pmed.1001931.s003.docx]

**S1 Table. Original study’s design and analysis plan and deviations from this plan for final study report**

| **Analysis plan** | **Current study** | **Justification** |
| --- | --- | --- |
| Defined the cohort as adolescents who are 10-15 years old in 2002 (i.e. the cohort highlighted in red on page 4 of the analysis plan). | Defined the cohort as adolescents who were 10-19 years old in 1997-2011 (i.e. the cohort highlighted in green on page 4 of the analysis plan). | We initially decided to use the cohort highlighted in red and to incorporate information from admissions during the 5 years before the index admission into covariates, and to look at outcomes during the 5 years after the index admission. However, we later acknowledged that many clinicians will vary in the amount of information available about previous admissions and therefore risks based on this covariate would not be very useful for identification of high-risk adolescents, compared with absolute 10-year risks in a much larger sample (which we could analyse if we used the cohort highlighted in green). This longer follow-up and larger sample size was a particular priority for us is as death is a rare outcome. |
| Define index admissions as all unplanned (emergency) admissions for injury between 10 and 19 years old. | Define index admissions as randomly selected emergency admissions for injury between 10 and 19 years old – one per adolescent. | We decided that it would be simpler for analysis and interpretation of results to randomly select an index admission than to use all emergency admissions for injury. Approximately 10% of adolescents had more than one emergency admission for injury during adolescence. As a sensitivity analysis, we carried out the main analyses with two randomly selected index admissions per adolescent (i.e., emergency admissions for injury between 10 and 19 years old; only 2% of adolescents had three or more), accounting for clustering of multiple admissions within individuals by using a shared frailty term in time-to-event models. The results were very similar to those when randomly selecting one index admission, e.g., the age-adjusted hazard ratio for death in girls was 1.84 (95% CI: 1.69 to 2.00), similar to that for our analyses in the current article (where the hazard ratio was 1.61, 95% confidence interval 1.43 to 1.82). |
| Include re-admissions for injury and adversity-related injury as outcomes. | Did not include re-admissions for injury and adversity-related injury as outcomes. | We carried out the main analyses for the outcomes of re-admission for injury and re-admission for adversity-related injury: there were similar associations between adversity-related/accident-related injury and these outcomes as between adversity-related/accident-related injury and death and emergency re-admissions (i.e. an increased risk of outcome by 1.5 to 2 times for adversity-related vs. accident-related). However, we felt that including full sets of results for re-admissions for injury and for adversity-related injury diluted the results for the final report, and so did not include them. Instead, we provided numbers of re-admissions for injury and for adversity-related injury in one of the article’s tables (Table A2 in S1 Text). |
| Defined drug/alcohol-related injury as any record for injury with a code mentioning drugs or alcohol, “with the exception of Y91 (‘Evidence of alcohol involvement determined by level of intoxication’) and Z72.1 (‘alcohol use’), which is only considered misuse when the subject is younger than 18 years old.” | Included Y91 and Z72.1 in the definition of drug/alcohol-related injury, even if adolescent was 18 years old or older. | During the review of a previous publication using these code lists,[1] we decided that any alcohol use, regardless of age, should be considered misuse if coupled with an emergency admission and injury. |
