# Supplementary material for: 10-y Risks of Death and Emergency Re-admission in Adolescents Hospitalised with Violent, Drug- or Alcohol-Related, or Self-Inflicted Injury: A Population-Based Cohort Study
Source: PLoS Med. 2015 Dec 29;12(12):e1001931. doi: 10.1371/journal.pmed.1001931 (PMC4699823; doi:10.1371/journal.pmed.1001931)
Supplement: S2 Table — (DOC) [file pmed.1001931.s004.doc]

**S2 Table. Derivation of 1-10 year risks of death in the general adolescent population.**

We derived the following table using the freely-available Excel document ‘England, National Life-tables, 1980-1982 to 2011-2013 (Excel sheet, 920kB)’: <http://www.ons.gov.uk/ons/publications/re-reference-tables.html?edition=tcm%3A77-365199>.

|  |  | **Pr(death at given age and within given time period)** | | | | | | | | | | |
| --- | --- | --- | --- | --- | --- | --- | --- | --- | --- | --- | --- | --- |
| **Sex** | **Age** | **1997-1999** | **1998-2000** | **1999-2001** | **2000-2002** | **2001-2003** | **2002-2004** | **2003-2005** | **2004-2006** | **2005-2007** | **2006-2008** | **2007-2009** |
| Girls | 10 | 0.000124 | 0.000110 | 0.000104 | 0.000096 | 0.000099 | 0.000105 | 0.000093 | 0.000079 | 0.000091 | 0.000084 | 0.000088 |
|  | 11 | 0.000113 | 0.000120 | 0.000121 | 0.000102 | 0.000100 | 0.000082 | 0.000089 | 0.000086 | 0.000093 | 0.000089 | 0.000089 |
|  | 12 | 0.000143 | 0.000132 | 0.000116 | 0.000107 | 0.000128 | 0.000135 | 0.000138 | 0.000116 | 0.000114 | 0.000098 | 0.000098 |
|  | 13 | 0.000135 | 0.000121 | 0.000103 | 0.000105 | 0.000101 | 0.000114 | 0.000114 | 0.000124 | 0.000118 | 0.000116 | 0.000109 |
|  | 14 | 0.000143 | 0.000141 | 0.000134 | 0.000151 | 0.000154 | 0.000153 | 0.000134 | 0.000123 | 0.000125 | 0.000108 | 0.000111 |
|  | 15 | 0.000204 | 0.000163 | 0.000172 | 0.000148 | 0.000151 | 0.000145 | 0.000146 | 0.000153 | 0.000137 | 0.000143 | 0.000135 |
|  | 16 | 0.000254 | 0.000247 | 0.000222 | 0.000239 | 0.000234 | 0.000226 | 0.000214 | 0.000212 | 0.000197 | 0.000169 | 0.000165 |
|  | 17 | 0.000302 | 0.000281 | 0.000274 | 0.000256 | 0.000257 | 0.000239 | 0.000225 | 0.000229 | 0.000238 | 0.000232 | 0.000210 |
|  | 18 | 0.000302 | 0.000322 | 0.000314 | 0.000307 | 0.000268 | 0.000260 | 0.000258 | 0.000262 | 0.000258 | 0.000257 | 0.000243 |
|  | 19 | 0.000289 | 0.000302 | 0.000287 | 0.000306 | 0.000301 | 0.000331 | 0.000314 | 0.000293 | 0.000252 | 0.000246 | 0.000237 |
| Boys | 10 | 0.000146 | 0.000137 | 0.000141 | 0.000127 | 0.000110 | 0.000104 | 0.000109 | 0.000123 | 0.000100 | 0.000101 | 0.000089 |
|  | 11 | 0.000160 | 0.000138 | 0.000132 | 0.000138 | 0.000144 | 0.000128 | 0.000118 | 0.000121 | 0.000128 | 0.000112 | 0.000100 |
|  | 12 | 0.000171 | 0.000161 | 0.000172 | 0.000156 | 0.000156 | 0.000142 | 0.000158 | 0.000151 | 0.000140 | 0.000115 | 0.000106 |
|  | 13 | 0.000188 | 0.000174 | 0.000175 | 0.000195 | 0.000198 | 0.000180 | 0.000163 | 0.000167 | 0.000171 | 0.000154 | 0.000139 |
|  | 14 | 0.000249 | 0.000223 | 0.000220 | 0.000218 | 0.000223 | 0.000210 | 0.000201 | 0.000193 | 0.000187 | 0.000170 | 0.000157 |
|  | 15 | 0.000275 | 0.000246 | 0.000240 | 0.000258 | 0.000260 | 0.000271 | 0.000248 | 0.000236 | 0.000232 | 0.000228 | 0.000246 |
|  | 16 | 0.000448 | 0.000424 | 0.000374 | 0.000381 | 0.000363 | 0.000358 | 0.000331 | 0.000333 | 0.000332 | 0.000337 | 0.000308 |
|  | 17 | 0.000594 | 0.000561 | 0.000567 | 0.000550 | 0.000538 | 0.000524 | 0.000520 | 0.000532 | 0.000520 | 0.000495 | 0.000468 |
|  | 18 | 0.000791 | 0.000782 | 0.000781 | 0.000784 | 0.000756 | 0.000695 | 0.000662 | 0.000623 | 0.000588 | 0.000555 | 0.000533 |
|  | 19 | 0.000844 | 0.000818 | 0.000818 | 0.000781 | 0.000738 | 0.000656 | 0.000647 | 0.000651 | 0.000641 | 0.000615 | 0.000586 |

We derived estimates for 1, 2,… 10 –year risks of death for a cohort of 10-19 year olds in 1997-1999 (thus 10-year estimates were available for the entire cohort). For example, the 1-year risk of death for a 10 year old in 1997-1999 was estimated as Pr(death at 10 years old in 1997-1999 or at 11 years old in 1998-2000) = 0.000124 + 0.000120.
