# Supplementary material for: 10-y Risks of Death and Emergency Re-admission in Adolescents Hospitalised with Violent, Drug- or Alcohol-Related, or Self-Inflicted Injury: A Population-Based Cohort Study
Source: PLoS Med. 2015 Dec 29;12(12):e1001931. doi: 10.1371/journal.pmed.1001931 (PMC4699823; doi:10.1371/journal.pmed.1001931)
Supplement: S1 Text — (DOC) [file pmed.1001931.s005.doc]

**S1 Text. Additional tables**

**Table A1. Numbers of adolescents with an index emergency admission for adversity- or accident- related injury and a** chronic condition, by type of condition

|  | **Girls** | | | |  | **Boys** | | | |
| --- | --- | --- | --- | --- | --- | --- | --- | --- | --- |
|  | **Adversity-related injury** | | **Accident-related injury** | |  | **Adversity-related injury** | | **Accident-related injury** | |
| **Total with history of a chronic condition** | 27,922 | (100.0) | 18,934 | (100.0) |  | 21,161 | (100.0) | 49,436 | (100.0) |
| **Type of chronic condition** |  |  |  |  |  |  |  |  |  |
| Mental health/behavioural | 9,348 | (33.5) | 1,702 | (9.0) |  | 6,984 | (33.0) | 6,063 | (12.3) |
| Cancer/blood disorders | 677 | (2.4) | 799 | (4.2) |  | 693 | (3.3) | 2,049 | (4.1) |
| Chronic infections | 58 | (0.2) | 50 | (0.3) |  | 116 | (0.5) | 164 | (0.3) |
| Respiratory | 11,213 | (40.2) | 9,452 | (49.9) |  | 8,429 | (39.8) | 27,411 | (55.4) |
| Metabolic/endocrine/digestive/renal/genitourinary | 4,475 | (16.0) | 2,421 | (12.8) |  | 2,619 | (12.4) | 4,912 | (9.9) |
| Musculoskeletal/skin | 1,730 | (6.2) | 2,608 | (13.8) |  | 1,127 | (5.3) | 4,403 | (8.9) |
| Neurological | 4,537 | (16.2) | 4,708 | (24.9) |  | 4,063 | (19.2) | 9,804 | (19.8) |
| Cardiovascular | 266 | (1.0) | 273 | (1.4) |  | 232 | (1.1) | 656 | (1.3) |
| Non-specific* | 207 | (0.7) | 428 | (2.3) |  | 176 | (0.8) | 776 | (1.6) |

*Lack of expected normal physiological development, feeding difficulties and mismanagement, ‘attention to gastronomy’, palliative care, holiday relief care, ‘gastrostomy status’, dependence on wheelchair.

**Table A2. Characteristics of deaths and emergency re-admissions within ten years following discharge from index admission.**

|  | **Girls** | | | |  | **Boys** | | | | |
| --- | --- | --- | --- | --- | --- | --- | --- | --- | --- | --- |
| **Variable, units** | **Adversity-related injury** | | **Accident-related injury** | |  | **Adversity-related injury** | | | **Accident-related injury** | |
| Number (column %) | 181,926 | (100.0) | 166,462 | (100.0) |  | 151,083 | | (100.0) | 483,356 | (100.0) |
| Follow-up in years, Median (IQR) | 6.8 | (3.3, 10.0) | 7.5 | (3.9, 10.0) |  | 7.0 | | (3.6, 10.0) | 7.7 | (4.1, 10.0) |
| Length of stay in days, Median (IQR) | 1 | (1, 1) | 1 | (1, 2) |  | 1 | | (1, 2) | 1 | (1, 1) |
| Died, n (per 1,000) | 873 | (4.8) | 439 | (2.6) |  | 1,542 | | (10.2) | 1,928 | (4.0) |
| Death related to injury, n (% who died) | 688 | (78.8) | 166 | (37.8) |  | 1,226 | | (79.5) | 1,314 | (68.2) |
| Time to death in years, Median (IQR) b | 3.4 | (1.4, 6.1) | 4.1 | (1.9, 6.5) |  | 3.3 | | (1.1, 5.9) | 3.1 | (1.3, 5.7) |
| Had an emergency re-admission, n (%) | 77,101 | (42.4) | 44,790 | (26.9) |  | 46,284 | | (30.6) | 108,812 | (22.5) |
| 1 | 34,846 | (19.2) | 25,479 | (15.3) |  | 26,008 | | (17.2) | 75,160 | (15.5) |
| 2+ | 42,255 | (23.2) | 19,311 | (11.6) |  | 20,276 | | (13.4) | 33,652 | (7.0) |
| 2 | 16,449 | (9.0) | 8,894 | (5.3) |  | 9,495 | | (6.3) | 19,925 | (4.1) |
| 3-5 | 17,112 | (9.4) | 7,367 | (4.4) |  | 7,519 | | (5.0) | 10,907 | (2.3) |
| 6+ | 8,694 | (4.8) | 3,050 | (1.8) |  | 3,262 | | (2.2) | 2,820 | (0.6) |
| Had an emergency re-admission for injury, n  (% who had an emergency re-admission) | 34,356 | (44.6) | 13,120 | (29.3) |  | 29,976 | | (64.8) | 59,970 | (55.1) |
| Total number of emergency re-admissions, n | 227,385 | . | 102,095 | . |  | 108,839 | . | | 182,731 | . |
| Total number of emergency re-admissions for injury, n  (% of all emergency re-admissions) | 67,084 | (29.5) | 18,415 | (18.0) |  | 53,887 | | (49.5) | 79,925 | (43.7) |
| Time to 1^st^ emergency re-admission in days, Median (IQR) | 586 | (179, 1,370) | 930 | (273, 1,898) |  | 750 | | (220, 1,596) | 933 | (262, 1,907) |
| Time from 1^st^-2^nd^ emergency re-admission in days, Median (IQR) | 299 | (75, 798) | 347 | (78, 926) |  | 345 | | (91, 895) | 430 | (98,1,084) |
| Time from 2^nd^-3^rd^ emergency re-admission in days, Median (IQR) | 205 | (50, 594) | 214 | (49, 592) |  | 224 | | (58, 622) | 256 | (62, 687) |

IQR = Inter-quartile Range

*Some adolescents were not followed up for the full ten years, e.g., adolescents admitted in 2011.

**Table A3. Numbers/risks of death within 1, 5, and 10 years of discharge from index admission.**

| **Girls** |  | **At discharge** |  | **1 year following discharge** | | | |  | **5 years following discharge** | | | |  | **10 years following discharge** | | | |
| --- | --- | --- | --- | --- | --- | --- | --- | --- | --- | --- | --- | --- | --- | --- | --- | --- | --- |
| **Age-group**  **Type of injury at index** |  | **Adolescents** |  | **Followed up** | **Died** | **Cumulative risk per 1,000 (95% CI)** | |  | **Followed up** | **Died** | **Cumulative risk per 1,000 (95% CI)** | |  | **Followed up** | **Died** | **Cumulative risk per 1,000 (95% CI)** | |
| 10-14 years |  |  |  |  |  |  |  |  |  |  |  |  |  |  |  |  |  |
| Adversity-related |  | 47,926 |  | 44,604 | 30 | 0.6 | (0.5, 0.9) |  | 31,981 | 95 | 2.4 | (1.9, 2.9) |  | 15,448 | 155 | 4.8 | (4.1, 5.6) |
| Any violent* |  | 3,931 |  | 3,650 | 1 | 0.3 | (0.0, 1.9) |  | 2,521 | 8 | 2.4 | (1.2, 4.9) |  | 1,111 | 12 | 4.3 | (2.4, 7.6) |
| Any drug/alcohol-related* |  | 41,996 |  | 39,225 | 28 | 0.7 | (0.5, 1.0) |  | 28,551 | 82 | 2.3 | (1.9, 2.9) |  | 14,026 | 135 | 4.7 | (3.9, 5.6) |
| Any self-inflicted* |  | 32,335 |  | 29,706 | 19 | 0.6 | (0.4, 1.0) |  | 20,501 | 53 | 2.0 | (1.5, 2.6) |  | 9,298 | 99 | 5.0 | (4.1, 6.2) |
| Accident-related |  | 103,215 |  | 96,671 | 39 | 0.4 | (0.3, 0.5) |  | 70,480 | 145 | 1.6 | (1.4, 1.9) |  | 35,772 | 211 | 2.8 | (2.5, 3.3) |
| 15-17 years |  |  |  |  |  |  |  |  |  |  |  |  |  |  |  |  |  |
| Adversity-related |  | 84,605 |  | 78,004 | 79 | 1.0 | (0.8, 1.2) |  | 52,230 | 235 | 3.4 | (2.9, 3.8) |  | 24,882 | 364 | 6.7 | (6.0, 7.5) |
| Any violent* |  | 5,277 |  | 4,931 | 2 | 0.4 | (0.1, 1.6) |  | 3,388 | 11 | 2.7 | (1.5, 4.9) |  | 1,466 | 26 | 8.1 | (5.4, 12.0) |
| Any drug/alcohol-related* |  | 77,240 |  | 71,230 | 74 | 1.0 | (0.8, 1.2) |  | 47,888 | 208 | 3.2 | (2.8, 3.7) |  | 23,104 | 320 | 6.4 | (5.7, 7.2) |
| Any self-inflicted* |  | 63,589 |  | 58,107 | 63 | 1.0 | (0.8, 1.3) |  | 37,742 | 182 | 3.5 | (3.0, 4.1) |  | 17,456 | 272 | 6.8 | (6.0, 7.7) |
| Accident-related |  | 36,624 |  | 34,470 | 20 | 0.6 | (0.4, 0.9) |  | 24,217 | 84 | 2.7 | (2.2, 3.3) |  | 11,679 | 122 | 5.0 | (4.2, 6.1) |
| 18-19 years |  |  |  |  |  |  |  |  |  |  |  |  |  |  |  |  |  |
| Adversity-related |  | 49,395 |  | 45,329 | 89 | 1.9 | (1.5, 2.3) |  | 28,975 | 246 | 6.0 | (5.3, 6.8) |  | 13,813 | 354 | 11.1 | (10.0, 12.4) |
| Any violent* |  | 4,075 |  | 3,808 | 3 | 0.7 | (0.2, 2.3) |  | 2,551 | 10 | 2.8 | (1.5, 5.2) |  | 1,191 | 16 | 6.1 | (3.6, 10.5) |
| Any drug/alcohol-related* |  | 44,822 |  | 41,003 | 81 | 1.9 | (1.5, 2.3) |  | 26,122 | 221 | 6.0 | (5.2, 6.8) |  | 12,504 | 321 | 11.2 | (10.0, 12.6) |
| Any self-inflicted* |  | 35,969 |  | 32,976 | 74 | 2.1 | (1.7, 2.7) |  | 20,668 | 201 | 6.8 | (5.9, 7.8) |  | 9,737 | 280 | 11.9 | (10.5, 13.5) |
| Accident-related |  | 26,623 |  | 24,920 | 26 | 1.0 | (0.7, 1.5) |  | 17,082 | 77 | 3.4 | (2.7, 4.3) |  | 8,234 | 106 | 5.7 | (4.7, 6.9) |
| All |  |  |  |  |  |  |  |  |  |  |  |  |  |  |  |  |  |
| Adversity-related |  | 181,926 |  | 167,937 | 198 | 1.1 | (1.0, 1.3) |  | 113,186 | 576 | 3.8 | (3.5, 4.1) |  | 54,143 | 873 | 7.3 | (6.8, 7.8) |
| Any violent* |  | 13,283 |  | 12,389 | 6 | 0.5 | (0.2, 1.0) |  | 8,459 | 29 | 2.6 | (1.8, 3.8) |  | 3,767 | 54 | 6.4 | (4.8, 8.4) |
| Any drug/alcohol-related* |  | 164,058 |  | 151,458 | 183 | 1.2 | (1.0, 1.3) |  | 102,561 | 511 | 3.7 | (3.4, 4.1) |  | 49,634 | 776 | 7.2 | (6.7, 7.7) |
| Any self-inflicted* |  | 131,893 |  | 120,789 | 156 | 1.2 | (1.1, 1.4) |  | 78,911 | 436 | 4.0 | (3.7, 4.4) |  | 36,491 | 651 | 7.7 | (7.1, 8.4) |
| Accident-related |  | 166,462 |  | 156,061 | 85 | 0.5 | (0.4, 0.6) |  | 111,779 | 306 | 2.1 | (1.9, 2.4) |  | 55,685 | 439 | 3.7 | (3.4, 4.1) |

**Table A3 (continued)**

| **Boys** |  | **At discharge** |  | **1 year following discharge** | | | |  | **5 years following discharge** | | | |  | **10 years following discharge** | | | |
| --- | --- | --- | --- | --- | --- | --- | --- | --- | --- | --- | --- | --- | --- | --- | --- | --- | --- |
| **Age-group**  **Type of injury at index** |  | **Adolescents** |  | **Followed up** | **Died** | **Cumulative risk per 1,000 (95% CI)** | |  | **Followed up** | **Died** | **Cumulative risk per 1,000 (95% CI)** | |  | **Followed up** | **Died** | **Cumulative risk per 1,000 (95% CI)** | |
| 10-14 years |  |  |  |  |  |  |  |  |  |  |  |  |  |  |  |  |  |
| Adversity-related |  | 24,301 |  | 22,923 | 13 | 0.6 | (0.3, 0.9) |  | 17,147 | 75 | 3.6 | (2.9, 4.6) |  | 8,838 | 146 | 9.2 | (7.8, 10.9) |
| Any violent* |  | 10,561 |  | 9,946 | 4 | 0.4 | (0.1, 1.1) |  | 7,269 | 22 | 2.4 | (1.6, 3.7) |  | 3,440 | 42 | 6.6 | (4.7, 9.0) |
| Any drug/alcohol-related* |  | 12,927 |  | 12,237 | 8 | 0.6 | (0.3, 1.3) |  | 9,426 | 49 | 4.4 | (3.3, 5.9) |  | 5,182 | 98 | 11.1 | (9.0, 13.6) |
| Any self-inflicted* |  | 6,641 |  | 6,207 | 2 | 0.3 | (0.1, 1.2) |  | 4,568 | 21 | 3.9 | (2.6, 6.0) |  | 2,332 | 45 | 10.5 | (7.8, 14.2) |
| Accident-related |  | 259,862 |  | 243,402 | 75 | 0.3 | (0.2, 0.4) |  | 177,108 | 381 | 1.7 | (1.6, 1.9) |  | 88,129 | 747 | 4.5 | (4.2, 4.8) |
| 15-17 years |  |  |  |  |  |  |  |  |  |  |  |  |  |  |  |  |  |
| Adversity-related |  | 57,706 |  | 53,619 | 97 | 1.7 | (1.4, 2.1) |  | 37,006 | 380 | 7.9 | (7.1, 8.7) |  | 17,827 | 551 | 14.3 | (13.1, 15.6) |
| Any violent* |  | 27,177 |  | 25,679 | 21 | 0.8 | (0.5, 1.2) |  | 17,993 | 107 | 4.7 | (3.9, 5.6) |  | 8,141 | 158 | 8.7 | (7.3, 10.2) |
| Any drug/alcohol-related* |  | 32,281 |  | 29,421 | 77 | 2.5 | (2.0, 3.1) |  | 20,120 | 274 | 10.4 | (9.2, 11.7) |  | 10,106 | 398 | 18.9 | (17.0, 20.9) |
| Any self-inflicted* |  | 17,747 |  | 16,281 | 55 | 3.2 | (2.5, 4.2) |  | 10,745 | 190 | 13.1 | (11.4, 15.1) |  | 5,407 | 265 | 22.5 | (19.8, 25.5) |
| Accident-related |  | 137,044 |  | 129,506 | 99 | 0.7 | (0.6, 0.9) |  | 93,420 | 419 | 3.6 | (3.3, 4.0) |  | 43,892 | 660 | 7.1 | (6.5, 7.7) |
| 18-19 years |  |  |  |  |  |  |  |  |  |  |  |  |  |  |  |  |  |
| Adversity-related |  | 69,076 |  | 63,427 | 186 | 2.8 | (2.4, 3.2) |  | 42,347 | 561 | 9.8 | (9.0, 10.6) |  | 20,519 | 845 | 19.1 | (17.8, 20.5) |
| Any violent* |  | 32,981 |  | 30,925 | 56 | 1.7 | (1.3, 2.3) |  | 21,267 | 176 | 6.2 | (5.4, 7.2) |  | 9,689 | 260 | 11.9 | (10.4, 13.5) |
| Any drug/alcohol-related* |  | 40,326 |  | 35,981 | 142 | 3.7 | (3.1, 4.3) |  | 23,164 | 407 | 12.6 | (11.5, 13.9) |  | 11,632 | 616 | 25.1 | (23.1, 27.3) |
| Any self-inflicted* |  | 20,352 |  | 18,679 | 89 | 4.5 | (3.7, 5.6) |  | 12,114 | 258 | 15.5 | (13.7, 17.5) |  | 6,334 | 394 | 30.4 | (27.4, 33.7) |
| Accident-related |  | 86,450 |  | 82,223 | 89 | 1.1 | (0.9, 1.3) |  | 59,269 | 338 | 4.5 | (4.1, 5.0) |  | 28,385 | 521 | 8.7 | (8.0, 9.5) |
| All |  |  |  |  |  |  |  |  |  |  |  |  |  |  |  |  |  |
| Adversity-related |  | 151,083 |  | 139,969 | 296 | 2.0 | (1.8, 2.3) |  | 96,500 | 1016 | 8.0 | (7.6, 8.6) |  | 47,184 | 1542 | 15.6 | (14.8, 16.4) |
| Any violent* |  | 70,719 |  | 66,550 | 81 | 1.2 | (0.9, 1.5) |  | 46,529 | 305 | 5.0 | (4.5, 5.7) |  | 21,270 | 460 | 9.8 | (8.9, 10.8) |
| Any drug/alcohol-related* |  | 85,534 |  | 77,639 | 227 | 2.8 | (2.4, 3.2) |  | 52,710 | 730 | 10.4 | (9.7, 11.2) |  | 26,920 | 1112 | 20.3 | (19.1, 21.6) |
| Any self-inflicted* |  | 44,740 |  | 41,167 | 146 | 3.4 | (2.9, 4.0) |  | 27,426 | 469 | 12.7 | (11.6, 14.0) |  | 14,073 | 704 | 24.1 | (22.3, 26.1) |
| Accident-related |  | 483,356 |  | 455,131 | 263 | 0.6 | (0.5, 0.6) |  | 329,797 | 1138 | 2.8 | (2.6, 2.9) |  | 160,406 | 1928 | 6.0 | (5.7, 6.3) |

**Table A4. Relative risks of death and re-admission, with and without adjustment for potential confounding factors.**

|  |  | **Hazard ratio for death (95% Confidence Interval)** | | | | | | | |
| --- | --- | --- | --- | --- | --- | --- | --- | --- | --- |
|  | **Variables at index** | **Unadjusted** | | **Adjusted for age** | | **Adjusted for age, chronic condition status** | | **Adjusted for age, chronic condition status, ethnicity, deprivation** | |
| Girls | Accident-related (vs. Accident-related) injury | 1.93 | (1.72, 2.16) | 1.61 | (1.43, 1.82) | 1.52 | (1.34, 1.71) | 1.48 | (1.31, 1.67) |
|  | Age-group (vs. 10-14 years): 15-17 years | . | . | 1.46 | (1.27, 1.69) | 1.39 | (1.20, 1.60) | 1.39 | (1.20, 1.60) |
|  | 18-19 years | . | . | 2.32 | (2.01, 2.68) | 2.05 | (1.78, 2.37) | 2.05 | (1.77, 2.36) |
|  | Chronic condition (vs. No) | . | . | . | . | 4.51 | (4.03, 5.04) | 4.45 | (3.98, 4.98) |
|  | Ethnicity (vs. White): Black | . | . | . | . | . | . | 0.99 | (0.68, 1.43) |
|  | Asian | . | . | . | . | . | . | 1.08 | (0.80, 1.46) |
|  | Mixed | . | . | . | . | . | . | 0.60 | (0.30, 1.20) |
|  | Other | . | . | . | . | . | . | 0.66 | (0.39, 1.12) |
|  | Missing | . | . | . | . | . | . | 0.85 | (0.71, 1.01) |
|  | Deprivation quintile (vs. Least deprived):  2^nd^ least deprived | . | . | . | . | . | . | 1.05 | (0.85, 1.30) |
|  | Middle quintile | . | . | . | . | . | . | 1.05 | (0.85, 1.29) |
|  | 2^nd^ most deprived | . | . | . | . | . | . | 1.20 | (0.99, 1.45) |
|  | Most deprived | . | . | . | . | . | . | 1.24 | (1.03, 1.49) |
|  | Missing | . | . | . | . | . | . | 0.20 | (0.06, 0.63) |
| Boys | Accident-related (vs. Accident-related) injury | 2.70 | (2.53, 2.89) | 2.13 | (1.98, 2.29) | 2.01 | (1.87, 2.16) | 1.92 | (1.79, 2.07) |
|  | Age-group (vs. 10-14 years): 15-17 years | . | . | 1.68 | (1.54, 1.84) | 1.72 | (1.58, 1.88) | 1.73 | (1.58, 1.89) |
|  | 18-19 years | . | . | 2.16 | (1.98, 2.37) | 2.23 | (2.03, 2.43) | 2.23 | (2.04, 2.44) |
|  | Chronic condition (vs. No) | . | . | . | . | 3.07 | (2.84, 3.32) | 3.01 | (2.79, 3.25) |
|  | Ethnicity (vs. White): Black | . | . | . | . | . | . | 1.03 | (0.83, 1.27) |
|  | Asian | . | . | . | . | . | . | 0.90 | (0.72, 1.11) |
|  | Mixed | . | . | . | . | . | . | 0.76 | (0.49, 1.18) |
|  | Other | . | . | . | . | . | . | 0.73 | (0.54, 0.98) |
|  | Missing | . | . | . | . | . | . | 0.79 | (0.73, 0.86) |
|  | Deprivation quintile (vs. Least deprived):  2^nd^ least deprived | . | . | . | . | . | . | 1.24 | (1.08, 1.42) |
|  | Middle quintile | . | . | . | . | . | . | 1.29 | (1.13, 1.47) |
|  | 2^nd^ most deprived | . | . | . | . | . | . | 1.44 | (1.27, 1.63) |
|  | Most deprived | . | . | . | . | . | . | 1.65 | (1.46, 1.85) |
|  | Missing | . | . | . | . | . | . | 0.54 | (0.36, 0.82) |

**Table A4 (continued)**

|  |  | **Hazard ratio for emergency re-admission (95% Confidence Interval)** | | | | | | | |
| --- | --- | --- | --- | --- | --- | --- | --- | --- | --- |
|  | **Variables at index** | **Unadjusted** | | **Adjusted for age** | | **Adjusted for age, chronic condition status** | | **Adjusted for age, chronic condition status, ethnicity, deprivation** | |
| Girls | Accident-related (vs. Accident-related) injury | 1.90 | (1.88, 1.93) | 1.76 | (1.74, 1.79) | 1.74 | (1.72, 1.77) | 1.67 | (1.65, 1.70) |
|  | Age-group (vs. 10-14 years): 15-17 years | . | . | 1.22 | (1.20, 1.23) | 1.20 | (1.18, 1.22) | 1.19 | (1.17, 1.20) |
|  | 18-19 years | . | . | 1.29 | (1.27, 1.31) | 1.26 | (1.24, 1.28) | 1.26 | (1.24, 1.28) |
|  | Chronic condition (vs. No) | . | . | . | . | 1.81 | (1.79, 1.84) | 1.73 | (1.70, 1.75) |
|  | Ethnicity (vs. White): Black | . | . | . | . | . | . | 0.79 | (0.76, 0.82) |
|  | Asian | . | . | . | . | . | . | 0.77 | (0.75, 0.80) |
|  | Mixed | . | . | . | . | . | . | 0.98 | (0.93, 1.03) |
|  | Other | . | . | . | . | . | . | 0.70 | (0.67, 0.73) |
|  | Missing | . | . | . | . | . | . | 0.18 | (0.17, 0.18) |
|  | Deprivation quintile (vs. Least deprived):  2^nd^ least deprived | . | . | . | . | . | . | 1.07 | (1.05, 1.10) |
|  | Middle quintile | . | . | . | . | . | . | 1.14 | (1.12, 1.17) |
|  | 2^nd^ most deprived | . | . | . | . | . | . | 1.23 | (1.20, 1.25) |
|  | Most deprived | . | . | . | . | . | . | 1.33 | (1.31, 1.36) |
|  | Missing | . | . | . | . | . | . | 0.20 | (0.17, 0.22) |
| Boys | Accident-related (vs. Accident-related) injury | 1.52 | (1.50, 1.53) | 1.41 | (1.39, 1.43) | 1.38 | (1.37, 1.40) | 1.35 | (1.34, 1.37) |
|  | Age-group (vs. 10-14 years): 15-17 years | . | . | 1.14 | (1.13, 1.15) | 1.15 | (1.13, 1.16) | 1.15 | (1.13, 1.16) |
|  | 18-19 years | . | . | 1.26 | (1.24, 1.27) | 1.27 | (1.25, 1.29) | 1.29 | (1.27, 1.30) |
|  | Chronic condition (vs. No) | . | . |  |  | 1.72 | (1.70, 1.75) | 1.62 | (1.60, 1.65) |
|  | Ethnicity (vs. White): Black | . | . | . | . | . | . | 0.79 | (0.76, 0.82) |
|  | Asian | . | . | . | . | . | . | 0.83 | (0.80, 0.86) |
|  | Mixed | . | . | . | . | . | . | 0.94 | (0.89, 0.99) |
|  | Other | . | . | . | . | . | . | 0.72 | (0.69, 0.75) |
|  | Missing | . | . | . | . | . | . | 0.26 | (0.25, 0.26) |
|  | Deprivation quintile (vs. Least deprived):  2^nd^ least deprived | . | . | . | . | . | . | 1.07 | (1.05, 1.09) |
|  | Middle quintile | . | . | . | . | . | . | 1.15 | (1.12, 1.17) |
|  | 2^nd^ most deprived | . | . | . | . | . | . | 1.24 | (1.22, 1.27) |
|  | Most deprived | . | . | . | . | . | . | 1.37 | (1.34, 1.39) |
|  | Missing | . | . | . | . | . | . | 0.22 | (0.20, 0.24) |

All variables were entered simultaneously into a Cox regression model (multi-variable results).

**Table A5. Numbers/risks of emergency re-admission within 1, 5, and 10 years of discharge from index admission.**

| **Girls** |  | **At discharge** |  | **1 year since discharge** | | | |  | **5 years since discharge** | | | |  | **10 years since discharge** | | | |
| --- | --- | --- | --- | --- | --- | --- | --- | --- | --- | --- | --- | --- | --- | --- | --- | --- | --- |
| **Age-group**  **Type of injury at index** |  | **Adolescents** |  | **Followed up** | **Re-admitted** | **Cumulative risk per 100 (95% CI)** | |  | **Followed up** | **Re-admitted** | **Cumulative risk per 100 (95% CI)** | |  | **Followed up** | **Re-admitted** | **Cumulative risk per 100 (95% CI)** | |
| 10-14 years |  |  |  |  |  |  |  |  |  |  |  |  |  |  |  |  |  |
| Adversity-related |  | 47,926 |  | 38,165 | 6,921 | 14.8 | (14.5, 15.2) |  | 20,608 | 15,926 | 37.6 | (37.1, 38.1) |  | 8,203 | 19,879 | 52.7 | (52.2, 53.3) |
| Any violent* |  | 3,931 |  | 3,215 | 472 | 12.4 | (11.4, 13.4) |  | 1,690 | 1,202 | 35.3 | (33.7, 37.0) |  | 605 | 1,521 | 51.4 | (49.4, 53.4) |
| Any drug/alcohol-related* |  | 41,996 |  | 33,619 | 5,995 | 14.7 | (14.3, 15.0) |  | 18,406 | 13,966 | 37.4 | (36.9, 37.9) |  | 7,446 | 17,502 | 52.5 | (51.9, 53.1) |
| Any self-inflicted* |  | 32,335 |  | 24,865 | 5,254 | 16.8 | (16.4, 17.2) |  | 12,608 | 11,461 | 40.6 | (40.0, 41.2) |  | 4,720 | 13,939 | 55.6 | (54.9, 56.3) |
| Accident-related |  | 103,215 |  | 90,283 | 6,777 | 6.7 | (6.5, 6.8) |  | 57,631 | 17,015 | 18.8 | (18.6, 19.1) |  | 24,730 | 24,911 | 33.1 | (32.8, 33.5) |
| 15-17 years |  |  |  |  |  |  |  |  |  |  |  |  |  |  |  |  |  |
| Adversity-related |  | 84,605 |  | 65,369 | 13,567 | 16.5 | (16.3, 16.8) |  | 31,727 | 30,141 | 41.1 | (40.7, 41.4) |  | 12,754 | 35,902 | 54.7 | (54.3, 55.2) |
| Any violent* |  | 5,277 |  | 4,224 | 750 | 14.6 | (13.6, 15.6) |  | 2,078 | 1,895 | 40.9 | (39.5, 42.4) |  | 760 | 2,246 | 54.1 | (52.4, 55.8) |
| Any drug/alcohol-related* |  | 77,240 |  | 59,841 | 12,209 | 16.3 | (16.1, 16.6) |  | 29,136 | 27,341 | 40.8 | (40.5, 41.2) |  | 11,866 | 32,662 | 54.6 | (54.1, 55.0) |
| Any self-inflicted* |  | 63,589 |  | 48,142 | 10,769 | 17.5 | (17.2, 17.8) |  | 22,329 | 23,238 | 42.6 | (42.2, 43.1) |  | 8,750 | 27,351 | 56.2 | (55.7, 56.7) |
| Accident-related |  | 36,624 |  | 31,163 | 3,470 | 9.7 | (9.4, 10.0) |  | 17,648 | 9,079 | 28.3 | (27.8, 28.8) |  | 7,377 | 11,533 | 41.2 | (40.5, 41.8) |
| 18-19 years |  |  |  |  |  |  |  |  |  |  |  |  |  |  |  |  |  |
| Adversity-related |  | 49,395 |  | 36,701 | 9,290 | 19.4 | (19.0, 19.8) |  | 16,989 | 18,741 | 43.5 | (43.0, 44.0) |  | 7,056 | 21,320 | 54.6 | (54.0, 55.2) |
| Any violent* |  | 4,075 |  | 3,187 | 659 | 16.6 | (15.5, 17.8) |  | 1,562 | 1,474 | 41.0 | (39.3, 42.6) |  | 639 | 1,710 | 52.5 | (50.6, 54.4) |
| Any drug/alcohol-related* |  | 44,822 |  | 33,200 | 8,400 | 19.4 | (19.0, 19.7) |  | 15,292 | 16,958 | 43.6 | (43.1, 44.1) |  | 6,361 | 19,285 | 54.7 | (54.1, 55.2) |
| Any self-inflicted* |  | 35,969 |  | 26,267 | 7,222 | 20.7 | (20.3, 21.2) |  | 11,784 | 14,208 | 45.4 | (44.8, 45.9) |  | 4,820 | 16,075 | 56.6 | (55.9, 57.2) |
| Accident-related |  | 26,623 |  | 22,182 | 2,901 | 11.2 | (10.8, 11.5) |  | 12,381 | 6,797 | 29.1 | (28.5, 29.7) |  | 5,318 | 8,346 | 40.7 | (40.0, 41.5) |
| All |  |  |  |  |  |  |  |  |  |  |  |  |  |  |  |  |  |
| Adversity-related |  | 181,926 |  | 140,235 | 29778 | 16.9 | (16.7, 17.0) |  | 69,324 | 64,808 | 40.8 | (40.6, 41.0) |  | 28,013 | 77,101 | 54.2 | (53.9, 54.5) |
| Any violent* |  | 13,283 |  | 10,626 | 1881 | 14.5 | (13.9, 15.2) |  | 5,328 | 4,571 | 39.3 | (38.4, 40.2) |  | 2,002 | 5,477 | 52.8 | (51.8, 53.9) |
| Any drug/alcohol-related* |  | 164,058 |  | 126,660 | 26604 | 16.7 | (16.5, 16.9) |  | 62,834 | 58,265 | 40.7 | (40.4, 40.9) |  | 25,673 | 69,449 | 54.1 | (53.8, 54.4) |
| Any self-inflicted* |  | 131,893 |  | 99,274 | 23245 | 18.2 | (18.0, 18.4) |  | 46,721 | 48,907 | 42.9 | (42.6, 43.2) |  | 18,290 | 57,365 | 56.2 | (55.8, 56.5) |
| Accident-related |  | 166,462 |  | 143,628 | 13148 | 8.1 | (7.9, 8.2) |  | 87,660 | 32,891 | 22.5 | (22.3, 22.8) |  | 37,425 | 44,790 | 36.1 | (35.8, 36.4) |

**Table A5 (continued)**

| **Boys** |  | **At discharge** |  | **1 year since discharge** | | | |  | **5 years since discharge** | | | |  | **10 years since discharge** | | | |
| --- | --- | --- | --- | --- | --- | --- | --- | --- | --- | --- | --- | --- | --- | --- | --- | --- | --- |
| **Age-group**  **Type of injury at index** |  | **Adolescents** |  | **Followed up** | **Re-admitted** | **Cumulative risk per 100 (95% CI)** | |  | **Followed up** | **Re-admitted** | **Cumulative risk per 100 (95% CI)** | |  | **Followed up** | **Re-admitted** | **Cumulative risk per 100 (95% CI)** | |
| 10-14 years |  |  |  |  |  |  |  |  |  |  |  |  |  |  |  |  |  |
| Adversity-related |  | 24,301 |  | 20,897 | 2,129 | 8.9 | (8.6, 9.3) |  | 13,121 | 5,274 | 24.3 | (23.7, 24.9) |  | 5,724 | 7,151 | 38.2 | (37.5, 39.0) |
| Any violent* |  | 10,561 |  | 9,252 | 7,29 | 7.1 | (6.6, 7.6) |  | 5,737 | 1,967 | 21.2 | (20.4, 22.1) |  | 2,280 | 2,724 | 35.2 | (34.1, 36.4) |
| Any drug/alcohol-related* |  | 12,927 |  | 11,031 | 1,260 | 9.9 | (9.4, 10.5) |  | 7,096 | 3,046 | 26.0 | (25.2, 26.8) |  | 3,315 | 4,119 | 39.9 | (38.9, 40.9) |
| Any self-inflicted* |  | 6,641 |  | 5,427 | 8,20 | 12.7 | (11.9, 13.5) |  | 3,243 | 1,829 | 30.8 | (29.6, 32.0) |  | 1,398 | 2,365 | 45.3 | (43.9, 46.8) |
| Accident-related |  | 259,862 |  | 227,489 | 16,759 | 6.6 | (6.5, 6.7) |  | 147,782 | 38,762 | 16.8 | (16.7, 17.0) |  | 64,240 | 54,292 | 28.3 | (28.1, 28.6) |
| 15-17 years |  |  |  |  |  |  |  |  |  |  |  |  |  |  |  |  |  |
| Adversity-related |  | 57,706 |  | 48,560 | 5,452 | 9.7 | (9.4, 9.9) |  | 27,525 | 13,523 | 27.1 | (26.7, 27.5) |  | 11,394 | 17,349 | 40.3 | (39.8, 40.8) |
| Any violent* |  | 27,177 |  | 23,993 | 1,781 | 6.7 | (6.4, 7.0) |  | 13,971 | 5,433 | 23.1 | (22.5, 23.6) |  | 5,400 | 7,267 | 36.7 | (36.0, 37.4) |
| Any drug/alcohol-related* |  | 32,281 |  | 26,050 | 3,662 | 11.7 | (11.4, 12.1) |  | 14,435 | 8,311 | 30.0 | (29.5, 30.6) |  | 6,251 | 10,436 | 43.2 | (42.5, 43.9) |
| Any self-inflicted* |  | 17,747 |  | 13,980 | 2,509 | 14.6 | (14.0, 15.1) |  | 7,337 | 5,229 | 34.1 | (33.4, 34.9) |  | 3,222 | 6,369 | 47.0 | (46.1, 47.9) |
| Accident-related |  | 137,044 |  | 121,002 | 8,936 | 6.6 | (6.5, 6.8) |  | 75,558 | 23,588 | 19.6 | (19.4, 19.8) |  | 30,953 | 32,326 | 31.8 | (31.5, 32.1) |
| 18-19 years |  |  |  |  |  |  |  |  |  |  |  |  |  |  |  |  |  |
| Adversity-related |  | 69,076 |  | 56,328 | 7,736 | 11.5 | (11.3, 11.8) |  | 30,077 | 18,131 | 30.5 | (30.1, 30.8) |  | 12,813 | 21,784 | 41.5 | (41.0, 42.0) |
| Any violent* |  | 32,981 |  | 28,463 | 2,636 | 8.2 | (7.9, 8.5) |  | 15,994 | 7,370 | 25.7 | (25.2, 26.3) |  | 6,427 | 9,168 | 36.8 | (36.2, 37.5) |
| Any drug/alcohol-related* |  | 40,326 |  | 31,169 | 5,317 | 13.7 | (13.4, 14.1) |  | 15,643 | 11,540 | 34.0 | (33.4, 34.5) |  | 6,905 | 13,554 | 45.0 | (44.4, 45.6) |
| Any self-inflicted* |  | 20,352 |  | 15,449 | 3,521 | 17.8 | (17.3, 18.4) |  | 7,551 | 7,087 | 39.9 | (39.2, 40.7) |  | 3,490 | 8,171 | 50.8 | (49.9, 51.6) |
| Accident-related |  | 86,450 |  | 76,022 | 6,514 | 7.7 | (7.5, 7.8) |  | 46,494 | 17,338 | 22.6 | (22.3, 22.9) |  | 19,984 | 22,194 | 33.1 | (32.7, 33.5) |
| All |  |  |  |  |  |  |  |  |  |  |  |  |  |  |  |  |  |
| Adversity-related |  | 151,083 |  | 125,785 | 15,317 | 10.4 | (10.2, 10.6) |  | 70,723 | 36,928 | 28.2 | (27.9, 28.4) |  | 29,931 | 46,284 | 40.5 | (40.2, 40.9) |
| Any violent* |  | 70,719 |  | 61,708 | 5,146 | 7.4 | (7.2, 7.6) |  | 35,699 | 14,770 | 24.0 | (23.7, 24.4) |  | 14,107 | 19,159 | 36.6 | (36.1, 37.0) |
| Any drug/alcohol-related* |  | 85,534 |  | 68,250 | 10,239 | 12.4 | (12.2, 12.6) |  | 37,174 | 22,897 | 31.2 | (30.9, 31.5) |  | 16,471 | 28,109 | 43.5 | (43.1, 44.0) |
| Any self-inflicted* |  | 44,740 |  | 34,856 | 6,850 | 15.8 | (15.4, 16.1) |  | 18,131 | 14,145 | 36.2 | (35.8, 36.7) |  | 8,110 | 16,905 | 48.5 | (47.9, 49.1) |
| Accident-related |  | 483,356 |  | 424,513 | 32,209 | 6.8 | (6.7, 6.8) |  | 269,834 | 79,688 | 18.7 | (18.5, 18.8) |  | 115,177 | 108,812 | 30.2 | (30.0, 30.3) |

Adolescents with records of any self-inflicted, any violent, or any drug/alcohol-related injury, respectively, at their index admission, are not mutually exclusive.

**Table A6. Relative risks of recurrent emergency re-admissions* within ten years following discharge from index admission.**

| **Sex, Model**  **Type of injury at index (vs. Accident-related injury)** |  | **Order of emergency re-admission after discharge from index**  Age-adjusted Hazard Ratio (95% Confidence Interval) | | | | | | | | | | | | | |
| --- | --- | --- | --- | --- | --- | --- | --- | --- | --- | --- | --- | --- | --- | --- | --- |
|  |  | **First** | |  | **Second** | |  | **Third** | |  | **Fourth** | |  | **Fifth** | |
| Girls, Model I |  |  |  |  |  |  |  |  |  |  |  |  |  |  |  |
| Adversity-related injury |  | 1.76 | (1.74, 1.79) |  | 2.09 | (2.05, 2.13) |  | 2.26 | (2.21, 2.32) |  | 2.35 | (2.28, 2.43) |  | 2.38 | (2.29, 2.46) |
| Girls, Model II |  |  |  |  |  |  |  |  |  |  |  |  |  |  |  |
| Violent injury only |  | 1.61 | (1.56, 1.66) |  | 1.95 | (1.87, 2.04) |  | 2.13 | (2.01, 2.25) |  | 2.24 | (2.09, 2.39) |  | 2.33 | (2.15, 2.53) |
| Drug/alcohol-related injury only |  | 1.47 | (1.44, 1.50) |  | 1.68 | (1.64, 1.73) |  | 1.82 | (1.76, 1.88) |  | 1.88 | (1.80, 1.97) |  | 1.86 | (1.76, 1.96) |
| Violent and drug/alcohol-related injury |  | 1.74 | (1.61, 1.88) |  | 2.07 | (1.86, 2.30) |  | 2.34 | (2.04, 2.68) |  | 2.10 | (1.76, 2.52) |  | 2.17 | (1.76, 2.69) |
| Self-inflicted injury only |  | 2.35 | (2.27, 2.44) |  | 3.00 | (2.85, 3.15) |  | 3.47 | (3.26, 3.69) |  | 3.91 | (3.63, 4.21) |  | 4.14 | (3.80, 4.52) |
| Self-inflicted and violent injury |  | 3.10 | (2.64, 3.63) |  | 3.43 | (2.74, 4.29) |  | 3.84 | (2.87, 5.13) |  | 4.44 | (3.15, 6.25) |  | 5.52 | (3.81, 8.01) |
| Self-inflicted and drug/alcohol-related injury |  | 1.86 | (1.83, 1.88) |  | 2.21 | (2.17, 2.25) |  | 2.37 | (2.31, 2.44) |  | 2.46 | (2.38, 2.54) |  | 2.48 | (2.39, 2.58) |
| Self-inflicted, violent and drug/alcohol-related injury |  | 2.21 | (1.97, 2.49) |  | 2.93 | (2.52, 3.41) |  | 3.31 | (2.74, 3.99) |  | 3.19 | (2.51, 4.05) |  | 3.04 | (2.27, 4.08) |
| Boys, Model I |  |  |  |  |  |  |  |  |  |  |  |  |  |  |  |
| Adversity-related injury |  | 1.41 | (1.39, 1.43) |  | 1.87 | (1.83, 1.90) |  | 2.29 | (2.23, 2.36) |  | 2.64 | (2.55, 2.74) |  | 2.94 | (2.81, 3.07) |
| Boys, Model II |  |  |  |  |  |  |  |  |  |  |  |  |  |  |  |
| Violent injury only |  | 1.16 | (1.14, 1.18) |  | 1.33 | (1.30, 1.37) |  | 1.44 | (1.38, 1.50) |  | 1.48 | (1.40, 1.56) |  | 1.54 | (1.43, 1.65) |
| Drug/alcohol-related injury only |  | 1.33 | (1.30, 1.36) |  | 1.75 | (1.70, 1.81) |  | 2.14 | (2.05, 2.24) |  | 2.48 | (2.34, 2.63) |  | 2.83 | (2.64, 3.03) |
| Violent and drug/alcohol-related injury |  | 1.29 | (1.24, 1.35) |  | 1.68 | (1.58, 1.78) |  | 2.03 | (1.86, 2.21) |  | 2.33 | (2.08, 2.60) |  | 2.55 | (2.23, 2.92) |
| Self-inflicted injury only |  | 1.84 | (1.76, 1.94) |  | 2.74 | (2.55, 2.94) |  | 3.63 | (3.30, 3.98) |  | 4.38 | (3.89, 4.93) |  | 4.69 | (4.06, 5.43) |
| Self-inflicted and violent injury |  | 2.02 | (1.60, 2.55) |  | 2.44 | (1.68, 3.54) |  | 3.50 | (2.18, 5.64) |  | 2.72 | (1.30, 5.72) |  | 3.87 | (1.74, 8.61) |
| Self-inflicted and drug/alcohol-related injury |  | 1.91 | (1.87, 1.94) |  | 2.85 | (2.78, 2.93) |  | 3.78 | (3.65, 3.91) |  | 4.59 | (4.39, 4.80) |  | 5.20 | (4.92, 5.50) |
| Self-inflicted, violent and drug/alcohol-related injury |  | 2.18 | (1.82, 2.61) |  | 3.87 | (3.05, 4.91) |  | 4.99 | (3.66, 6.80) |  | 6.31 | (4.32, 9.21) |  | 7.50 | (4.83, 11.64) |

*Time to each emergency re-admission was defined as the time since discharge from the index admission.

**Table A7. Ten-year risks of a) death and b) emergency re-admission, by chronic condition status and deprivation.**

a) Death, per 1,000 (95% Confidence Intervals)*

| **Sex** | **Status at index admission** | | **10-14y** | | | |  | **15-17y** | | | |  | **18-19y** | | | |
| --- | --- | --- | --- | --- | --- | --- | --- | --- | --- | --- | --- | --- | --- | --- | --- | --- |
|  |  |  | **Adversity-related** | | **Accident-related** | |  | **Adversity-related** | | **Accident-related** | |  | **Adversity-related** | | **Accident-related** | |
| Girls | No chronic condition | Least deprived | 3.3 | (1.8, 6.0) | 1.9 | (1.2, 3.0) |  | 4.8 | (3.2, 7.2) | 1.9 | (0.8, 4.6) |  | 6.3 | (3.7, 10.8) | 3.6 | (1.9, 6.7) |
|  |  | 2^nd^ least deprived | 9.6 | (4.3, 21.6) | 10.8 | (6.0, 19.5) |  | 19.6 | (11.5, 33.3) | 21.8 | (11.3, 42.0) |  | 28.2 | (16.3, 48.3) | 12.4 | (4.8, 32.2) |
|  |  | Middle deprived | 5.5 | (3.5, 8.7) | 1.9 | (1.2, 2.9) |  | 4.0 | (2.7, 5.9) | 2.1 | (1.1, 3.9) |  | 6.0 | (3.9, 9.3) | 3.0 | (1.6, 5.9) |
|  |  | 2^nd^ most deprived | 7.2 | (3.0, 17.4) | 13.6 | (8.4, 22.1) |  | 14.6 | (8.6, 25.0) | 18.7 | (8.7, 39.8) |  | 25.4 | (15.3, 42.2) | 23.7 | (11.8, 47.2) |
|  |  | Most deprived | 4.0 | (2.4, 6.5) | 1.2 | (0.7, 2.0) |  | 4.8 | (3.4, 6.6) | 3.3 | (1.7, 6.2) |  | 8.4 | (6.0, 11.7) | 3.0 | (1.6, 5.8) |
|  |  | Missing | 9.5 | (3.5, 26.0) | 16.4 | (10.9, 24.6) |  | 12.0 | (6.9, 20.6) | . |  |  | . |  | . |  |
|  | Chronic condition | Least deprived | 3.2 | (2.1, 4.9) | 1.8 | (1.2, 2.9) |  | 6.5 | (5.1, 8.3) | 3.4 | (1.9, 6.1) |  | 7.2 | (5.2, 10.0) | 3.9 | (2.1, 7.3) |
|  |  | 2^nd^ least deprived | 12.6 | (7.0, 22.6) | 17.0 | (11.2, 25.9) |  | 17.5 | (11.2, 27.3) | 22.0 | (12.2, 39.6) |  | 26.8 | (18.8, 38.1) | 33.5 | (19.2, 58.1) |
|  |  | Middle deprived | 4.5 | (3.3, 6.1) | 1.2 | (0.8, 1.9) |  | 5.3 | (4.2, 6.7) | 4.1 | (2.6, 6.4) |  | 10.6 | (8.6, 13.2) | 3.9 | (2.4, 6.5) |
|  |  | 2^nd^ most deprived | 10.9 | (6.1, 19.4) | 18.8 | (13.3, 26.4) |  | 19.6 | (14.3, 26.9) | 24.9 | (15.3, 40.4) |  | 27.6 | (20.5, 37.1) | 18.2 | (9.8, 33.5) |
|  |  | Most deprived | 2.9 | (0.4, 20.8) | . |  |  | 1.6 | (0.2, 11.0) | . |  |  | . |  | . |  |
|  |  | Missing | . |  | . |  |  | . |  | . |  |  | . |  | . |  |
| Boys | No chronic condition | Least deprived | 8.9 | (5.2, 15.4) | 2.2 | (1.7, 3.0) |  | 10.4 | (7.4, 14.7) | 4.5 | (3.5, 5.7) |  | 9.9 | (7.3, 13.4) | 4.5 | (3.2, 6.2) |
|  |  | 2^nd^ least deprived | 14.7 | (4.4, 48.5) | 9.9 | (6.7, 14.7) |  | 20.6 | (10.5, 40.5) | 14.9 | (9.7, 22.9) |  | 43.5 | (28.1, 67.1) | 23.5 | (13.7, 40.2) |
|  |  | Middle deprived | 6.1 | (3.4, 11.1) | 2.7 | (2.1, 3.4) |  | 10.6 | (7.7, 14.6) | 5.6 | (4.5, 6.9) |  | 17.2 | (13.7, 21.6) | 6.6 | (5.1, 8.6) |
|  |  | 2^nd^ most deprived | 17.7 | (8.2, 37.9) | 16.8 | (12.4, 22.7) |  | 25.9 | (14.9, 44.9) | 15.4 | (10.1, 23.5) |  | 40.2 | (25.8, 62.2) | 17.8 | (10.3, 30.6) |
|  |  | Most deprived | 7.3 | (4.3, 12.3) | 2.9 | (2.3, 3.7) |  | 11.7 | (9.0, 15.1) | 6.4 | (5.2, 7.9) |  | 14.9 | (12.1, 18.3) | 9.2 | (7.5, 11.4) |
|  |  | Missing | 10.4 | (4.3, 25.3) | 9.7 | (6.6, 14.2) |  | 17.4 | (9.5, 31.5) | . |  |  | . |  | . |  |
|  | Chronic condition | Least deprived | 8.0 | (5.3, 12.1) | 3.9 | (3.2, 4.7) |  | 14.2 | (11.6, 17.4) | 6.6 | (5.4, 8.1) |  | 16.6 | (13.9, 19.8) | 8.1 | (6.5, 10.1) |
|  |  | 2^nd^ least deprived | 13.5 | (6.7, 26.8) | 14.8 | (11.1, 19.8) |  | 30.4 | (21.4, 43.3) | 20.0 | (13.5, 29.5) |  | 43.3 | (32.4, 57.8) | 24.2 | (15.5, 37.8) |
|  |  | Middle deprived | 9.6 | (7.1, 13.0) | 4.6 | (3.9, 5.3) |  | 15.0 | (12.9, 17.4) | 7.0 | (5.9, 8.3) |  | 18.6 | (16.3, 21.1) | 9.1 | (7.6, 10.9) |
|  |  | 2^nd^ most deprived | 15.5 | (8.8, 27.3) | 23.3 | (18.8, 28.9) |  | 23.6 | (17.1, 32.7) | 24.1 | (17.8, 32.4) |  | 47.3 | (37.8, 59.1) | 30.7 | (21.6, 43.4) |
|  |  | Most deprived | . |  | 2.0 | (0.7, 5.2) |  | 6.4 | (2.6, 15.5) | . |  |  | 5.8 | (3.0, 11.2) | 2.8 | (0.9, 9.0) |
|  |  | Missing | . |  | . |  |  | . |  | . |  |  | . |  | . |  |

**Table A7 (continued)**

**b) Emergency re-admission, per 100 (95% Confidence Intervals**)

| **Sex** | **Status at index admission** | | **10-14y** | | | |  | **15-17y** | | | |  | **18-19y** | | | |
| --- | --- | --- | --- | --- | --- | --- | --- | --- | --- | --- | --- | --- | --- | --- | --- | --- |
|  |  |  | **Adversity-related** | | **Accident-related** | |  | **Adversity-related** | | **Accident-related** | |  | **Adversity-related** | | **Accident-related** | |
| Girls | No chronic condition | Least deprived | 45.3 | (43.6, 47.0) | 25.1 | (24.3, 26.0) |  | 46.7 | (45.4, 48.1) | 32.8 | (31.3, 34.3) |  | 46.1 | (44.3, 47.9) | 30.4 | (28.6, 32.2) |
|  |  | 2^nd^ least deprived | 48.8 | (47.2, 50.4) | 28.1 | (27.2, 28.9) |  | 49.1 | (47.9, 50.4) | 35.6 | (34.1, 37.2) |  | 48.2 | (46.5, 49.9) | 36.8 | (35.0, 38.7) |
|  |  | Middle deprived | 49.5 | (48.1, 51.0) | 30.6 | (29.7, 31.5) |  | 52.2 | (51.1, 53.3) | 37.9 | (36.4, 39.5) |  | 50.6 | (49.1, 52.1) | 35.4 | (33.7, 37.2) |
|  |  | 2^nd^ most deprived | 53.2 | (51.9, 54.4) | 34.2 | (33.4, 35.1) |  | 53.7 | (52.7, 54.6) | 41.6 | (40.1, 43.1) |  | 52.2 | (50.9, 53.5) | 41.1 | (39.3, 43.0) |
|  |  | Most deprived | 54.6 | (53.6, 55.6) | 38.3 | (37.6, 39.1) |  | 57.1 | (56.3, 57.9) | 47.8 | (46.4, 49.2) |  | 56.0 | (55.0, 57.1) | 48.2 | (46.5, 49.9) |
|  |  | Missing | 8.7 | (6.2, 12.3) | 6.1 | (4.7, 7.9) |  | 8.6 | (6.6, 11.0) | 5.0 | (3.4, 7.3) |  | 5.0 | (3.5, 7.1) | 4.5 | (2.9, 6.9) |
|  | Chronic condition | Least deprived | 59.6 | (55.3, 63.9) | 38.1 | (35.1, 41.3) |  | 63.0 | (59.9, 66.2) | 49.0 | (44.6, 53.7) |  | 65.1 | (61.3, 68.8) | 49.4 | (44.2, 54.8) |
|  |  | 2^nd^ least deprived | 65.0 | (60.9, 69.2) | 44.2 | (41.3, 47.2) |  | 66.7 | (63.9, 69.6) | 50.6 | (46.3, 55.1) |  | 66.8 | (63.2, 70.4) | 51.7 | (46.9, 56.7) |
|  |  | Middle deprived | 64.8 | (60.9, 68.6) | 47.2 | (44.1, 50.4) |  | 69.9 | (67.2, 72.6) | 55.3 | (51.0, 59.7) |  | 72.5 | (69.5, 75.4) | 54.3 | (49.7, 59.0) |
|  |  | 2^nd^ most deprived | 67.1 | (63.7, 70.5) | 51.1 | (48.3, 54.0) |  | 68.9 | (66.5, 71.3) | 59.7 | (55.8, 63.7) |  | 69.5 | (66.8, 72.1) | 60.7 | (56.3, 65.2) |
|  |  | Most deprived | 68.5 | (65.7, 71.4) | 54.0 | (51.6, 56.5) |  | 70.6 | (68.7, 72.6) | 62.7 | (59.1, 66.3) |  | 73.4 | (71.3, 75.4) | 64.0 | (60.1, 67.9) |
|  |  | Missing | 15.1 | (7.8, 28.2) | 15.9 | (8.3, 29.3) |  | 16.7 | (10.4, 26.3) | 16.7 | (7.6, 34.3) |  | 1.8 | (0.5, 7.0) | 3.7 | (0.9, 14.0) |
| Boys | No chronic condition | Least deprived | 29.4 | (27.3, 31.8) | 23.5 | (23.0, 24.0) |  | 32.2 | (30.7, 33.7) | 26.0 | (25.3, 26.7) |  | 32.0 | (30.6, 33.4) | 27.4 | (26.5, 28.4) |
|  |  | 2^nd^ least deprived | 34.5 | (32.3, 36.8) | 25.0 | (24.4, 25.5) |  | 35.1 | (33.7, 36.6) | 28.1 | (27.4, 28.9) |  | 36.9 | (35.5, 38.2) | 29.2 | (28.3, 30.2) |
|  |  | Middle deprived | 34.8 | (32.9, 36.9) | 26.6 | (26.1, 27.2) |  | 37.5 | (36.2, 38.9) | 30.2 | (29.5, 31.0) |  | 37.2 | (36.0, 38.4) | 31.1 | (30.2, 32.0) |
|  |  | 2^nd^ most deprived | 37.1 | (35.3, 38.8) | 28.7 | (28.2, 29.2) |  | 41.3 | (40.1, 42.5) | 33.0 | (32.3, 33.8) |  | 41.4 | (40.3, 42.5) | 33.9 | (33.0, 34.8) |
|  |  | Most deprived | 39.1 | (37.7, 40.4) | 31.2 | (30.7, 31.6) |  | 42.7 | (41.8, 43.7) | 36.3 | (35.6, 37.0) |  | 45.3 | (44.5, 46.2) | 38.0 | (37.2, 38.8) |
|  |  | Missing | 6.8 | (4.1, 11.2) | 5.9 | (5.0, 7.0) |  | 4.6 | (3.3, 6.4) | 6.1 | (4.9, 7.6) |  | 4.7 | (3.7, 5.9) | 6.5 | (5.3, 8.1) |
|  | Chronic condition | Least deprived | 45.6 | (40.1, 51.6) | 35.3 | (33.5, 37.2) |  | 46.4 | (42.0, 51.1) | 36.1 | (33.5, 38.7) |  | 54.9 | (50.4, 59.6) | 36.7 | (33.4, 40.1) |
|  |  | 2^nd^ least deprived | 54.6 | (48.9, 60.4) | 37.2 | (35.4, 39.1) |  | 48.2 | (44.2, 52.2) | 37.1 | (34.7, 39.7) |  | 50.8 | (47.0, 54.7) | 43.6 | (40.3, 47.1) |
|  |  | Middle deprived | 47.2 | (42.5, 52.1) | 37.0 | (35.2, 38.8) |  | 51.1 | (47.4, 55.0) | 39.6 | (37.1, 42.2) |  | 57.0 | (53.7, 60.4) | 44.9 | (41.8, 48.2) |
|  |  | 2^nd^ most deprived | 49.2 | (45.2, 53.4) | 41.0 | (39.3, 42.7) |  | 53.6 | (50.5, 56.9) | 45.1 | (42.5, 47.7) |  | 57.5 | (54.6, 60.3) | 48.2 | (45.0, 51.5) |
|  |  | Most deprived | 52.8 | (49.4, 56.3) | 44.9 | (43.3, 46.4) |  | 55.9 | (53.3, 58.5) | 48.6 | (46.3, 50.9) |  | 61.5 | (59.1, 63.8) | 52.3 | (49.5, 55.1) |
|  |  | Missing | 10.8 | (2.5, 39.9) | 12.3 | (7.9, 19.0) |  | 9.6 | (4.7, 19.0) | 8.8 | (4.3, 17.4) |  | 3.9 | (1.7, 8.5) | 12.2 | (7.0, 21.0) |

*Where there was not a sufficient number of deaths with which to calculate a cumulative risk, this is indicated by ‘.’

1. Herbert A, Gilbert R, González-Izquierdo A, Li L (2015) Violence, self-harm and drug or alcohol misuse in adolescents admitted to hospitals in England for injury: a retrospective cohort study. BMJ Open 5.
